# Pt Particles on a Dynamic TiO_2_ Support in Near-Ambient Conditions–Disentangling Size, Pressure, and Support Effects

**DOI:** 10.1021/jacs.5c14353

**Published:** 2025-10-17

**Authors:** Florian Kraushofer, Matthias Krinninger, Marina de la Higuera-Domingo, Lorenz Falling, Lukas Strauss, Sebastian Kaiser, Mohammad Salehi, Gaurav Anand, Virginia Pérez Dieste, Monika Blum, Barbara A. J. Lechner

**Affiliations:** † Functional Nanomaterials Group and Catalysis Research Center, Department of Chemistry, TUM School of Natural Sciences, 9184 Technical University of Munich, Garching 85748, Germany; ‡ ALBA Synchrotron Light Source, Carrer de la Llum 2-26, Cerdanyola del Vallès, Barcelona 08290, Spain; § Chemical Sciences Division and Advanced Light Source, 1666 Lawrence Berkeley National Laboratory, Berkeley, California 94720, United States; ∥ Institute for Advanced Study, Technical University of Munich, Garching 85748, Germany

## Abstract

Platinum particles on reducible oxides are known to form complex and highly dynamic catalyst systems at elevated pressures and temperatures, often adopting active structures that differ from those found at room temperature and under ultrahigh vacuum (UHV). Here, we study the oxidation and structural evolution of subnanometer Pt clusters and nanoparticles supported on rutile TiO_2_(110) across an oxygen pressure range from UHV to 0.1 mbar, using near-ambient pressure X-ray photoelectron spectroscopy (NAP-XPS), scanning tunneling microscopy (STM) under UHV and NAP conditions, and low-energy ion scattering (LEIS). Our results reveal distinct differences in oxidation behavior and thermal stability between Pt nanoparticles and clusters, which are further modulated by the support stoichiometry and oxygen pressure. Small Pt clusters become oxidized even at room temperature but are susceptible to accelerated sintering in 0.1 mbar O_2_ at elevated temperatures. In contrast, well-crystallized Pt nanoparticles on near-stoichiometric TiO_2_ show weaker oxidation. On a reduced, defective TiO_2_ support, Pt instead quickly becomes deeply buried by new titania layers, which are formed during support reoxidation. This process appears to result primarily from interactions of the support with the gas phase, unlike the classical, self-limited encapsulation that is induced by the strong metal–support interaction (SMSI). Finally, we address the full complexity of real catalysts in a direct side-by-side comparison of the single-crystalline model system with a Pt-loaded TiO_2_ powder catalyst (P25). We conclude that the stoichiometry of the model supports must be carefully chosen and controlled to accurately reproduce the expected state of powder supports during redox reactions.

## Graphical abstract

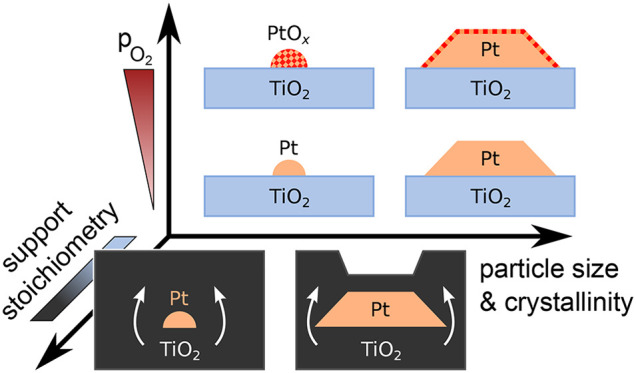

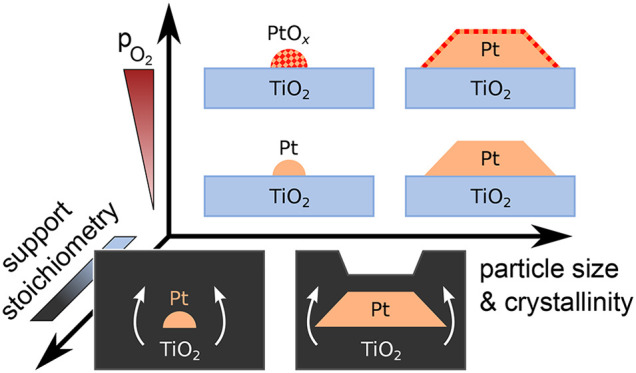

## 1 Introduction

Oxide-supported platinum particles feature among the most ubiquitous catalytic systems, most prominently in three-way automotive catalysts, where they are key to oxidizing CO and NO_*x*_. Understanding and thereby gaining the ability to improve such catalysts is a common goal, and has led to various attempts at elucidating the oxidation state of these Pt particles under working conditions. Historically, many studies have investigated the (111) surface of crystalline platinum, which is expected to be the dominant facet and still serves as a benchmark to this day.^1–3^ With the advent of near-ambient pressure (NAP) methods, the “pressure gap” between classical ultrahigh vacuum (UHV)-based surface-science studies and the elevated pressures can now often be closed to a significant degree.^4^ To extend the understanding gained from single-crystalline Pt(111) surfaces to oxide-supported Pt particles and further to real powder catalysts presents a major challenge, which is in turn described as the “complexity gap”.^4,5^ While the presence of other facets, edges and kink sites is still somewhat accessible to simple model studies,^6^ it is now widely accepted that the influence of oxide supports—in particular reducible oxide supports—must not be neglected.

Supports can sometimes participate directly in catalytic processes, providing sites for individual reaction steps or supplying oxygen through Mars-van-Krevelen-type mechanisms.^7,8^ Charge transfer to the support also affects the particles’ reactivity, which is sometimes described as an “electronic metal–support interaction” (EMSI).^9,10^ Similarly, the so-called “strong metal–support interaction” (SMSI) can change a nanoparticle’s capacity to adsorb molecules from the gas phase, which typically decreases sharply after the catalyst is heated under reducing conditions.^11,12^ Here, Pt on rutile titania (Pt/TiO_2_) is a prototypical example, where this SMSI effect is now understood to be due to encapsulation of the particles by a thin, substoichiometric TiO_*x*_ layer.^13–18^ This phenomenon has been investigated in depth for metal nanoparticles on many reducible oxides.^19–21^ In addition to the classical “encapsulation by SMSI”, other related phenomena have also been reported, most importantly de-encapsulation in a mixed reaction atmosphere and a nonclassical SMSI where a thin stoichiometric TiO_2_ layer overgrows the Pt in oxidizing atmospheres.^18,22–24^ A further key aspect in oxide-supported Pt catalysts is that small metal clusters (which we define here as particles smaller than 1 nm in size) often exhibit very different properties from larger nanoparticles. As bulk periodicity is lost at small sizes, so is the band structure, yielding a more molecule-like electronic configuration, which can lead to a very different and sometimes significantly higher reactivity than for more bulk-like, several nm large nanoparticles.^25,26^ Furthermore, the high surface-to-volume ratio and highly undercoordinated surface sites allow clusters to more easily restructure and incorporate oxygen.^27^

In oxygen-rich conditions, a key question concerns the active state of Pt during an ongoing reaction. Here, controversial reports can be found in the literature. On the one hand, it has been proposed that the most active phase for CO oxidation is an O-covered metallic Pt surface, rather than a surface or bulk oxide.^1,28,29^ On the other hand, exceptionally high CO oxidation activity has been reported for highly oxidized PtO_*x*_ species.^3,27,30^ Due to the large number of experimental parameters (sample complexity, pressure range, Pt particle size, and crystallinity, etc.), it is often difficult to directly compare results between the various studies. This has motivated us to investigate the state of supported Pt particles in oxygen by controlling and varying these parameters individually in a systematic, fundamental investigation.

This paper tackles the influence of an oxidative environment on TiO_2_-supported Pt clusters and nanoparticles. In this context, it is necessary to briefly review how bare rutile TiO_2_ interacts with gas phase oxygen, which has been investigated in much detail at low pressures, revealing the dominant bulk defects in reduced TiO_2–*x*_ are titanium interstitials (Ti_int_),^31^ up to a concentration of *x* ≈4 × 10^–4^, or one in 1250 unit cells.^32^ With sufficiently high near-surface reduction, the most common rutile (110) facet forms a (1 × 2) surface reconstruction.^33–35^ In our group, we have previously developed preparation recipes to reproducibly obtain single crystals with a well-defined stoichiometry: We distinguish between a “highly reduced” (HR-TiO_2_) and a near-stoichiometric or “low-reduced” (LR-TiO_2_) state. The HR-TiO_2_ samples are bulk-reduced to a level just before the onset of the (1 × 2) surface reconstruction, i.e. they contain the highest concentration of bulk defects that can be obtained without any long-range structural reorganization.^36^ When TiO_2_ is reoxidized, the excess bulk titanium interstitials diffuse to the surface and react with O_2_, ultimately forming new TiO_2_ terraces via several intermediate steps.^37,38^ It has also been shown previously that this oxidative layer growth is accelerated in the vicinity of Pd nanoparticles, and that these particles can be entirely covered by the newly grown TiO_2_ layers.^39^ In a previous study on Pt/TiO_2_(110), some of the present authors have found that Pt can also become buried when annealed in low oxygen pressures,^40^ though more rigorous control of support stoichiometry in the present study reveals that this behavior mainly depends on the availability of excess Ti interstitials.

In this work, we investigate the stability and evolution of Pt on rutile TiO_2_(110) as a function of oxygen pressure and particle size, using (NAP-)­STM and NAP-XPS, as well as low-energy ion scattering (LEIS) and near-edge X-ray absorption fine structure (NEXAFS) spectroscopies. Strong platinum oxidation is only observed at near-ambient pressures (0.1 mbar) of oxygen, and we find that small metal clusters interact with oxygen much more readily and more strongly than large nanoparticles, forming highly oxidized species. We also systematically control the support stoichiometry, distinguishing LR-TiO_2_ and HR-TiO_2_ samples as introduced previously.^36^ We show that this parameter is highly relevant to catalyst deactivation, as oxygen will react directly with HR-TiO_2–*x*_, leading to the deep, nonclassical burial of the metal particles in the oxide. In contrast, the well-known classical SMSI effect, i.e. encapsulation by a thin and reduced oxide film, is only observed on sufficiently reduced HR-TiO_2_ supports,^11,17^ but not on the LR-TiO_2_ samples.^36^ Similarly, we argue that the oxidative deep burial of Pt particles requires a large number of available Ti_int_. Based on observed and simulated TiO_2_ growth rates, we deduce that there has to be a rapid exchange of Ti_int_ with the bulk, with typical diffusion paths of several μm below the surface. In this context, we compare the single-crystalline supports to a Pt-loaded TiO_2_ powder catalyst (P25) and discuss the validity and limits of “model systems”. Nanoscale TiO_2_ powders grains have a much smaller bulk Ti_int_ reservoir, which means that the same process quickly leads to a full bulk reduction and reoxidation, thus coupling support stoichiometry to the chemical potential of the gas phase. Model single crystal studies allow disentangling these factors to identify how Pt particles are affected by the support stoichiometry and by the gas phase, respectively.

## 2 Experimental Methods

Single-crystalline TiO_2_(110) samples were acquired from SurfaceNet GmbH and subjected to cleaning cycles of sputtering (1 keV Ar^+^ ions) and annealing. HR-TiO_2_ and near-stoichiometric LR-TiO_2_ samples were obtained by automated preparation cycles, using the recipes discussed in detail elsewhere.^36^ Briefly, this includes a reoxidation step in 5 × 10^–6^ mbar O_2_ at 900 K after each sputtering step for both types of sample to avoid long-term drift of the bulk stoichiometry. The highly reduced samples are then annealed at 1100 K in UHV, while the near-stoichiometric samples are instead annealed at 1100 K in 5 × 10^–6^ mbar O_2_, then cooled down while keeping the oxygen chemical potential constant by decreasing the pressure simultaneously with the temperature. Pt clusters and nanoparticles were produced on these supports by physical vapor deposition of 0.05–0.2 monolayers (ML) respectively 7 ML Pt (Goodfellow, 99.95%) and sintering for 5 min at 600 K respectively 30 min at 1000–1200 K (1 K/s heating ramps) in UHV. We define 1 ML as one atom per TiO_2_(110) surface unit cell, i.e. 5.2 × 10^14^ cm^–2^. For comparative experiments with a powder sample, we use the same samples as in electron microscopy studies reported in the literature:^18,22^ TiO_2_ (Aeroxide P25, Acros Organics) was impregnated with tetraammineplatinum­(II) nitrate (99.995%, Sigma-Aldrich) dissolved in ultrapure Milli-Q water, resulting in a Pt weight loading of 2%. The sample was calcined at 200 °C in static air for 5 h, then heated under He flow (50 mL/min) in a tubular oven to 700 °C for 1 h (heating rate: 10 K/min). For NAP-XPS measurements, the powder was pressed into pellets and mounted like the single-crystal samples. Note that we cannot entirely rule out a substantial systematic error in the temperature readout for the pellet, with the sample being colder than measured, due to limited thermal conductivity.

(NAP-)­STM measurements were conducted in a UHV system consisting of two chambers with a base pressure of <1 × 10^–10^ mbar. One of the chambers houses an SPM Aarhus 150 NAP instrument (SPECS). STM was performed in constant-current mode, using electrochemically etched tungsten tips. Samples were sputtered (IQE 11, SPECS) and annealed using an electron-beam heater in the preparation chamber. Pt was deposited from an electron-beam evaporator (EBE-1, SPECS), using a quartz-crystal microbalance (OmniVac) to calibrate the deposition rate. Samples were mounted on stainless steel plates, and their temperature was measured during preparation and during experiments by a type K thermocouple pressed to the back of the sample by a spring. O_2_ gas was acquired from Westfalen AG (grade 5.0). STM images were corrected in ImageJ by row alignment along the slow-scan direction and subsequent plane subtraction. Apparent heights of Pt clusters were determined by evaluating their maximum apparent height with respect to the median height of the supporting TiO_2_ terrace. Mean apparent heights were averaged over 130–450 clusters, extracted from multiple STM images on different locations on the sample. Clusters at step edges were not included in the analysis, as they cannot be clearly assigned to one terrace.

The TiO_2_(110) samples for all NAP-XPS, LEIS and NEXAFS experiments were initially also prepared in the NAP-STM setup to obtain the desired support stoichiometry, then transferred in air to the respective facilities. There, they were cleaned by sputtering and annealing. Cleanliness was checked by XPS survey and C 1*s* spectra. Pt deposition was performed in situ in each setup, using a FOCUS EFM-3 and a SPECS EBE-4 evaporator, respectively.

LEIS and laboratory-based NAP-XPS measurements were performed in-house (base pressure <5 × 10^–10^ mbar). Pt coverage was controlled using as-deposited XPS spectra and calibrated against samples from the NAP-STM system. Samples were heated from the back using a laser heater (OsTech DioSource, 976 nm). XPS data were acquired with a PHOIBOS 150 NAP hemispherical analyzer (SPECS) with a 300 μm diameter aperture and a monochromated Al Kα X-ray source (μFOCUS 450 with XR-MC, SPECS). The same analyzer and a scannable ion source (IQE 12, SPECS) were used for LEIS (1025 eV He^+^ ions, 132.5° scattering angle).

Additional NAP-XPS measurements on supported Pt clusters were conducted at beamline 9.3.2 ^41,42^ of the Advanced Light Source (ALS) at the Lawrence Berkeley National Laboratory and NAP-XPS and NEXAFS measurements comparing Pt-loaded P25 powder samples and Pt nanoparticles on TiO_2_(110) single crystals were performed at the CIRCE beamline at ALBA Synchrotron. Pt deposition rates were estimated based on evaporator flux current, and coverages were later calibrated as discussed in the Supporting Information. All XPS spectra were measured with a beam energy *h*ν of 650 eV and referenced to the Ti 2*p* peak. NEXAFS was acquired by measuring the total electron yield on the sample, while varying the beam energy. At the ALS, a pyrolytic boron nitride heater was used for temperature control, and the temperature was monitored with a type K thermocouple mounted at the front side of the single crystals. At ALBA, a resistive button heater integrated in the sample holder was used to heat the sample, while the temperature was measured with a type K thermocouple clamped to the front side of the single crystals.

XPS binding energies were referenced to the Ti 2*p* peak set to 458.5 eV. Peaks were fitted with KolXPD using Shirley backgrounds. In all cases, the Ti 3*s* peak (which is close to the Pt 4*f* and can thus be measured with the same probing depth) as well as its plasmon were fitted with Voigt line shapes before Pt was deposited. These peak shapes, as well as the area and position of the plasmon peak relative to the main Ti 3*s* peak, were then kept constant. Pt 4*f* peaks were always fitted with a doublet of fixed 4:3 intensity ratio and 3.35 eV energy separation. Pt clusters and the oxidized components of Pt nanoparticles could be well-described by Voigt line shapes, while Doniach-Sunjic line shapes were used to fit the more bulk-like metallic components of Pt nanoparticles. The peak shape was initially left free to obtain the best fit to the as-sintered spectra, then kept fixed in fits for subsequent experimental conditions.

Pt quantification is based on the area ratios between the Pt 4*f* and Ti 3*s* peaks, which were always recorded at the same time, thus requiring no normalization. Spectra in the figures below are normalized for clarity, either to the low-energy background or to the Ti 3*s* peak area (specified in the figure captions). The Pt 4*f* to Ti 3*s* peak area ratios are based on numerical integrals of the raw data, as shown in Figures S3 and S6. Integration assumes a background with the start and end points set at the low and high binding energy side of the respective peaks. To minimize the influence of noise on these backgrounds, background support points were determined as the average of a number of neighboring data points. Shirley backgrounds were used when the high-binding-energy background is higher than the low-binding-energy background by more than the noise level (i.e., for most Pt nanoparticle data); otherwise, we default to linear backgrounds. Error bars were obtained by varying the integration ranges, as well as the averaging window for the background support points. The area of the Ti 3*s* plasmon, which overlaps the Pt 4*f* peak, is assumed to be a fixed fraction of the main Ti 3*s* peak; we therefore subtract it from the Pt 4*f*/Ti 3*s* area fraction, using the relative areas of the plasmon and main Ti 3*s* peaks determined before Pt deposition.

## 3 Results

We have performed comprehensive experiments controlling three independent parameters: particle size, O_2_ pressure, and support stoichiometry. To distinguish the individual effects, a systematic comparison of experiments is required, where parameters are varied one at a time. Therefore, in the following, we present four sections where the influence of two different pressure regimes on two Pt particle sizes, namely clusters and nanoparticles, are investigated. The influence of the TiO_2_(110) support stoichiometry is further discussed throughout. Finally, we address the full complexity of real systems by a direct side-by-side comparison of single-crystals and powder Pt/TiO_2_ samples and discuss the effect of particle crystallinity.

### 3.1 Pt Clusters in Low Oxygen Pressures

Figure 1 illustrates the simple case of O_2_ interacting with Pt clusters on reduced rutile TiO_2_(110). A typical STM image of the HR-TiO_2_(110) surface after depositing 0.05 ML Pt at room temperature (RT) and sintering at 600 K to form the clusters is shown in Figure 1a. The Pt clusters (some of which are marked by white arrows) are scattered on the surface, with a distribution of apparent heights around 4.0 ± 0.9 Å. Based on the cluster density analyzed over multiple STM images and the QCM-calibrated amount of deposited Pt, we can estimate that clusters contain on average ca. 9 Pt atoms. Interestingly, some of the clusters appear to be surrounded by a darker region (green arrows), possibly indicating that the surface is slightly etched during the sintering process. Furthermore, after sintering clusters at 600 K, we always find a high density of point defects (blue arrows), which we assign as the Ti_2_O_3_-type precursor species of the (1 × 2) reconstruction.^33–35^ These defects are commonly observed on rutile TiO_2_(110) surfaces when reducing or reoxidizing at low temperatures.^36–38^ They can be understood as a metastable feature formed when surface redox processes proceed faster than local structural rearrangement. In this case, the higher concentration of these defects than on the as-prepared surface suggests that the surface becomes slightly more reduced during the UHV sintering step.

**Figure 1:**
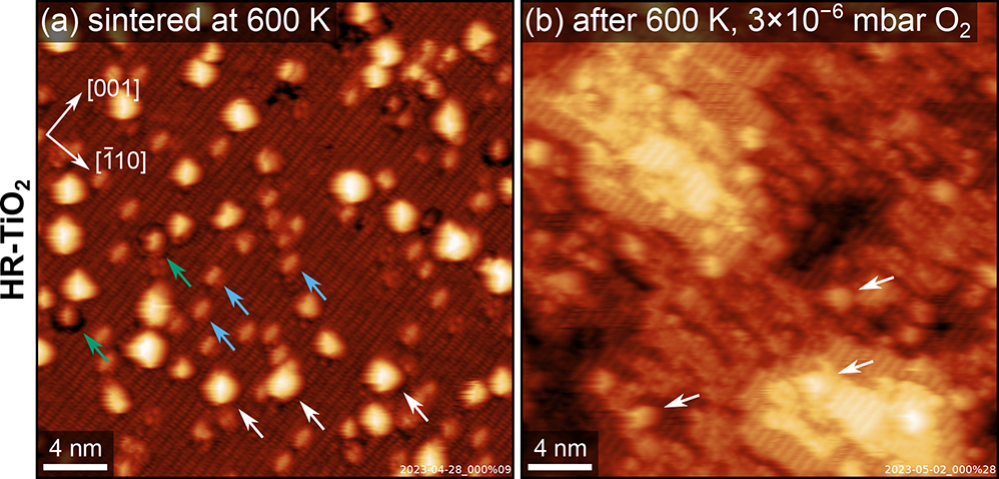
Pt clusters and oxidative layer growth on TiO_2_. STM images of HR-TiO_2_(110) (a) after depositing 0.05 ML Pt at RT and sintering at 600 K, and (b) after annealing at 600 K for 5 min in 3 × 10^–6^ mbar O_2_. White, green and blue arrows mark some Pt clusters, dark depressions and bright reduced point defects, respectively. Imaging conditions: RT, UHV, (a) *U*_b_ = 1.9 V, *I*_t_ = 0.1 nA, and (b) *U*_b_ = 1.8 V, *I*_t_ = 0.1 nA.

We then exposed the same sample to 3 × 10^–6^ mbar O_2_ and annealed it at 600 K for 5 min (1 K/s heating ramp). The results in Figure 1b show a distinct roughening of the surface, in analogy to what we have observed previously for the bare support.^36^ The Pt clusters are still visible (white arrows), but are embedded in newly grown TiO_2_ terraces. This terrace growth is a reoxidation of the reduced support via diffusion of bulk Ti_int_ to the surface and reaction with O_2_.^37,38^ Our group’s previous NAP-XPS investigation^40^ has shown that Pt does not become oxidized under these conditions, and that the new TiO_2_ layers completely overgrow the Pt clusters in a matter of minutes. The same growth of new TiO_2_ terraces is not observed on LR-TiO_2_.

### 3.2 Pt Clusters in Near-Ambient Oxygen Pressures

The behavior becomes more complex when moving to near-ambient oxygen pressure, as shown in Figure 2. It now also becomes important to control the support stoichiometry: We have shown previously that the growth of new TiO_2_ layers becomes primarily limited by the concentration of Ti_int_ in the bulk support,^36^ unlike at low pressure, where growth is limited by the availability of gas-phase O_2_.^37,38^ Figure 2a,e show Pt clusters after Pt deposition and 600 K UHV sintering on LR-TiO_2_ and HR-TiO_2_, respectively. We note that the clusters in Figure 2e are already more sintered compared to those in Figure 1a and more point defects are observed on the surface, caused by significantly longer annealing at the relevant temperatures during preceding STM experiments (not shown).

**Figure 2:**
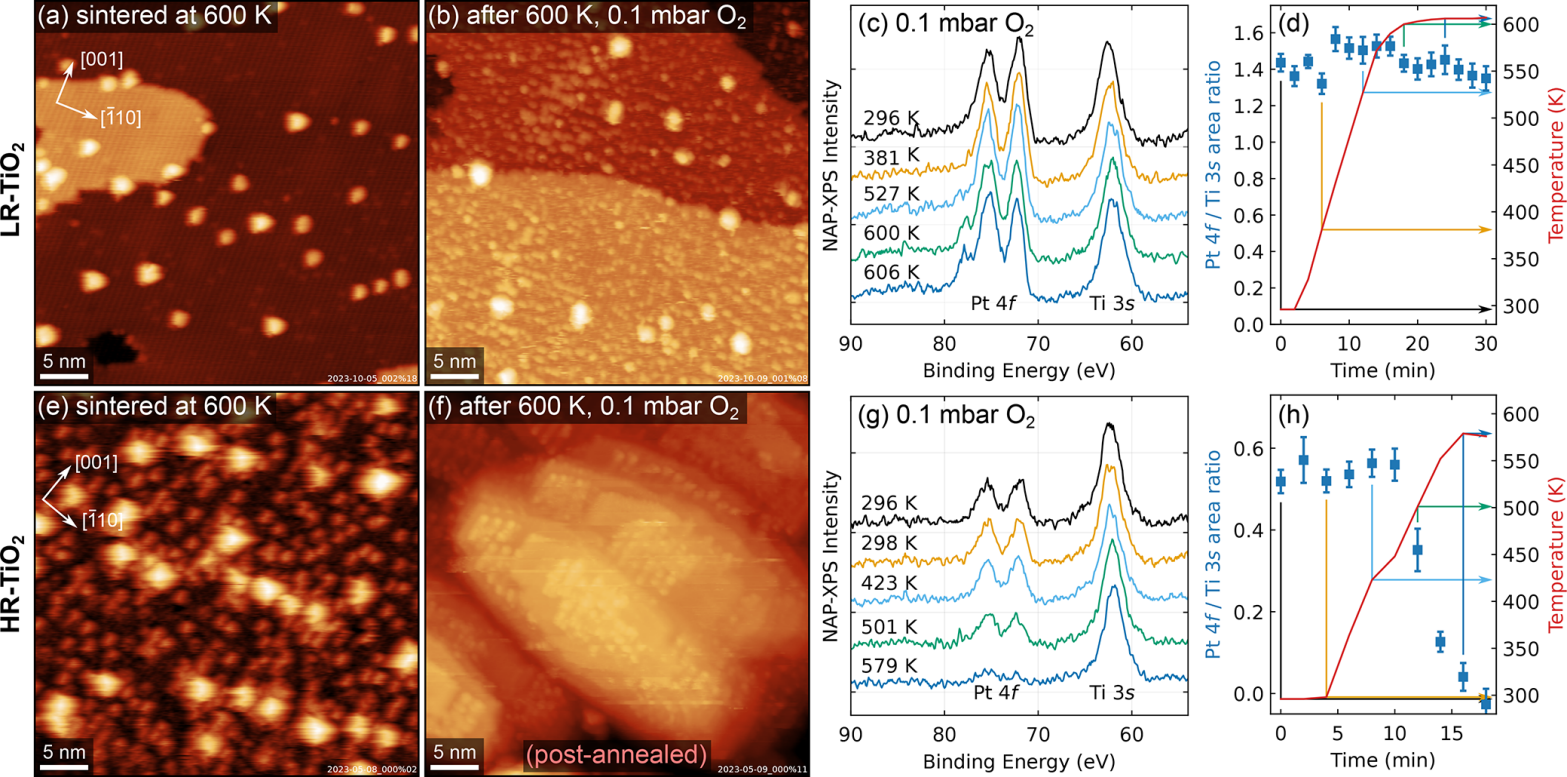
Effect of NAP O_2_ exposure on Pt clusters supported on (a–d) LR-TiO_2_ and (e–h) HR-TiO_2_, respectively. STM images of 0.05 ML Pt deposited on (a) LR-TiO_2_ and (e) HR-TiO_2_ show Pt clusters after sintering at 600 K. The same samples after (b) 20 min in 0.1 mbar O_2_ at 600 K, and (f) 30 min in 0.1 mbar O_2_ at 600 K, then postannealing at 800 K for 20 min in UHV to recover a sufficiently stable surface for STM. Imaging conditions: RT, UHV, (a) *U*_b_ = 1.7 V, *I*_t_ = 0.1 nA, (b) *U*_b_ = 2.0 V, *I*_t_ = 0.1 nA, (e) *U*_b_ = 1.6 V, *I*_t_ = 0.2 nA, and (f) *U*_b_ = 3.1 V, *I*_t_ = 0.1 nA. (c,g) NAP-XPS spectra (*h*ν = 650 eV, normalized to Ti 3*s* peak) of the Pt 4*f* and Ti 3*s* region from a separate experiment with equivalent sample preparation, acquired in 0.1 mbar O_2_ while heating from room temperature to 600 K. Note that the Pt coverage in the experiment in (c,d) is 0.2 ML. (d,h) Area ratios of the integrated Pt 4*f* and Ti 3*s* peaks (blue squares) as well as the temperature (red curve) as a function of time. Colored arrows indicate which spectra in (c,g) correspond to the respective data points.

Figure 2b,f show both samples after exposing them to 0.1 mbar O_2_ and annealing at 600 K. On LR-TiO_2_ (Figure 2b), the modification of the TiO_2_ support is essentially the same as was found in the absence of Pt,^36^ i.e. a variety of bright point defects appear due to oxidation of Ti_int_.^37,38,43^ The Pt clusters on LR-TiO_2_ are still clearly visible, though with decreased density and increased size, suggesting further sintering during the NAP O_2_ heating. Based on cluster density, the mean number of Pt atoms per cluster almost doubles, from ≈12 to ≈20. Meanwhile, their mean apparent height also increases slightly, from 3.9 ± 0.9 Å to 5.0 ± 1.4 Å. At this point, it should be noted that due to the more inhomogeneous, defect-rich appearance of the TiO_2_ support, we likely underestimate the apparent heights after O_2_ exposure, and possibly also under-count small clusters, which are difficult to distinguish from larger defects. Histograms of cluster height distributions before and after NAP O_2_ treatment for both the 0.05 ML coverage (Figure 2a,b) and 0.2 ML coverage (Figure S1) are shown in Figure S2, exhibiting a similar distribution for both coverages after the oxygen annealing step.

The changes upon heating in O_2_ are much more drastic on HR-TiO_2_ than on LR-TiO_2_. As with blank TiO_2_ supports, the surface roughens significantly, so much so that STM imaging directly after NAP O_2_ treatment was impossible.^36^ Figure 2f was recorded after postannealing the HR-TiO_2_ sample in UHV at 800 K for 20 min. Despite the postannealing treatment, the roughness is still significantly higher than is ever observed on an as-prepared sample. As discussed in prior work on the bare support, this is due to rapid reoxidation of the surface, where the rate of layer growth exceeds the rate of structural rearrangement into well-ordered (1 × 1) terraces.^36^ Notably, no signs of Pt clusters remain after this treatment—it appears as though they are completely overgrown by the support, which we will further corroborate below.

Corresponding NAP-XPS data were acquired under analogous experimental conditions. Note that in these experiments, the deposited amount of Pt was initially only estimated based on evaporator flux and later determined by SESSA^44,45^ simulations (see Supporting Information for details), giving 0.2 ML Pt on LR-TiO_2_ and 0.05 ML Pt on HR-TiO_2_. To exclude a possible coverage effect, we repeated the STM experiment with 0.2 ML Pt on LR-TiO_2_, shown in Figure S1, which yields comparable results. Selected NAP-XPS spectra of the Pt 4*f* and Ti 3*s* region acquired during the temperature ramp are shown in Figure 2c,g, and the full data sets (including Ti 2*p* peaks) are shown in Figure S3. Figure 2d,h show the area ratios of the integrated Pt 4*f* and Ti 3*s* peaks as a function of time throughout the experiment, with the applied temperature ramp superimposed in red. For a different visualization, the same data are plotted as a function of temperature in Figure S4.

The difference between LR-TiO_2_ and HR-TiO_2_ is again immediately apparent: While the Pt 4*f* area only decreases slightly over the entire time period on LR-TiO_2_, the platinum signal falls to zero in a matter of minutes on HR-TiO_2_ once the temperature reaches ≈500 K. This indicates that the Pt clusters become completely buried on this support, in good agreement with the STM image in Figure 2f. On LR-TiO_2_, the long-term behavior also fits well with STM. The slow decrease in Pt 4*f* signal after reaching 600 K can be understood as continued sintering in NAP O_2_, as was also observed in STM (Figures 2b and S1). Interestingly, the Pt 4*f* to Ti 3*s* peak area ratio increases from ≈1.3 to ≈1.6 when the sample is heated above ≈400 K, possibly due to a change in the particle shape. Furthermore, a strongly oxidized Pt species is formed under these conditions: The Pt 4*f*_5/2_ component develops a high binding energy side feature at ≈77.9 eV in Figure 2c (see the light blue, green and dark blue curves).

To illustrate the Pt oxidation on LR-TiO_2_ more clearly, Figure 3a,b shows Ti 2*p* and Pt 4*f* spectra from the same experiment, acquired before and after the data shown in Figure 2c. Directly after sintering (black curves in Figure 3), the Ti 2*p* peak exhibits a shoulder at ≈456.5 eV binding energy characteristic for Ti^3+^, as indicated by the arrow in Figure 3a. The Pt 4*f* data (Figure 3b) can be fitted well by a Voigt-shape doublet (peak intensity ratio 4:3, energy separation 3.35 eV) and a Shirley background. In the following, all Pt 4*f* peak positions refer to the Pt 4*f*_7/2_ peak, as the Pt 4*f*_5/2_ component is fully dependent. The gray peak at ≈75.7 eV corresponds to the Ti 3*s* plasmon, which was first fitted without constraints in spectra without Pt to obtain the peak shape, then linked to the Ti 3*s* peak at ≈62 eV after Pt was deposited.

**Figure 3:**
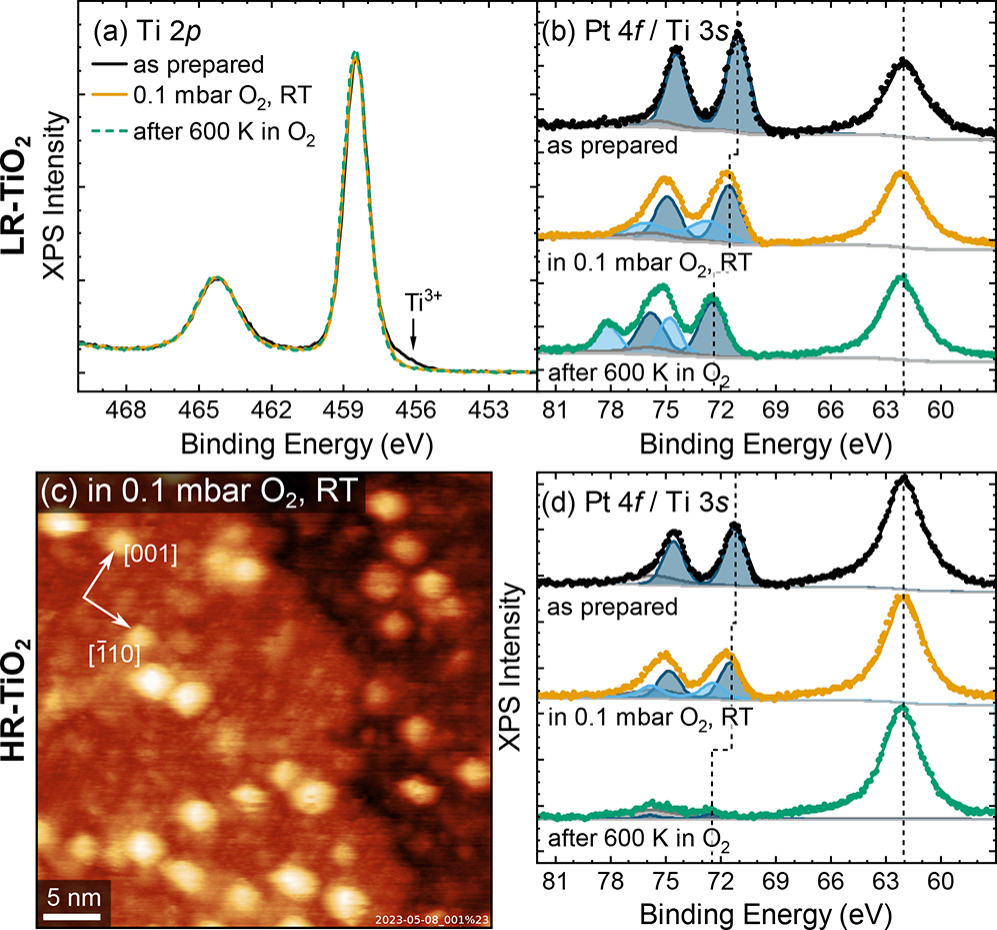
In situ measurements of the oxidation of Pt clusters on TiO_2_. XPS spectra (*h*ν = 650 eV, normalized to Ti 3*s* peak) for Pt clusters (a,b) on LR-TiO_2_ and (d) on HR-TiO_2_ show the Ti 2*p*, Pt 4*f* and Ti 3*s* regions at room temperature in UHV after sintering at 600 K (black), at room temperature in 0.1 mbar O_2_ (orange), and at room temperature in UHV after heating to 600 K in O_2_ (green). (c) In situ NAP-STM image of Pt clusters on HR-TiO_2_, acquired while exposing the sample shown in Figure 2e to 0.1 mbar O_2_ at room temperature, before heating to 600 K in O_2_. Imaging conditions: RT, 0.1 mbar O_2_, *U*_b_ = 1.6 V, *I*_t_ = 0.3 nA.

Introducing 0.1 mbar O_2_ at RT (orange curves in Figure 3) is sufficient to completely quench the Ti^3+^ signal, and introduces a broad, oxidized Pt 4*f* component (light blue) at 72.7 eV. The main Pt 4*f* peak (dark blue) also shifts from 71.1 to 71.5 eV. Here, all energies are referenced to the Ti 2*p* peak at 458.5 eV, which highlights the relative band alignment of Pt and the support. Interestingly, in the raw data, the main Pt peak position is constant, and all Ti and O peaks instead shift to lower binding energies when introducing oxygen (see Figure S3). This suggests that the observed shift is linked to a change in the support, likely modified band bending in the n-type TiO_2_ due to transfer of electrons to oxygen, and not just to O_2_ interacting with the Pt clusters. The same overall trends are seen in the Pt 4*f* spectra for Pt on HR-TiO_2_ (Figure 3d). However, after annealing at 600 K in 0.1 mbar O_2_ (green curves in Figure 3), the behavior on the two samples diverges. The main Pt component (dark blue) on LR-TiO_2_ shifts by another 0.9 to 72.4 eV, suggesting significant oxidation. The more oxidized component (light blue), which we tentatively assign as Pt^2+^, is significantly sharper than the one observed at room temperature, and shifted to 74.8 eV. These shifts are the same in the raw data, i.e. the observed Ti and O peak positions are unaffected by O_2_ annealing. Likewise, no changes are observed in the Ti 2*p* peak shape. On the HR-TiO_2_ support, the Pt intensity is almost entirely lost.

An in situ NAP-STM image of Pt on HR-TiO_2_, acquired at RT while exposing the sample shown in Figure 2e to 0.1 mbar O_2_, is shown in Figure 3c. In the NAP-XPS experiments, this would correspond to the orange data in Figure 3d. The Pt clusters appear qualitatively unchanged, but the TiO_2_ support has a much more diffuse appearance, and the abundant point defects seen in Figure 2e are less apparent, indicating that oxidation begins even at RT.

### 3.3 Pt Nanoparticles in Low Oxygen Pressures

Having explored the oxidation and burial of Pt clusters, we now turn to more bulk-like Pt nanoparticles. Again, it is instructive to first consider the case of low oxygen pressures. Figure 4 summarizes how annealing HR-TiO_2_ at low (10^–6^ mbar) oxygen pressure affects supported Pt nanoparticles. The STM image in Figure 4a shows Pt nanoparticles obtained by depositing 7 ML Pt and annealing at 1200 K. As reported previously, Pt nanoparticles on HR-TiO_2_ always exhibit an SMSI overlayer already after the initial sintering step, which at this particle size has a “pinwheel” structure,^15,16,36^ as seen in the inset to Figure 4a. Note that there is some similarity between this structure and the one reported for the Pt(111) surface oxide,^46^ but we can definitely exclude a Pt oxide at this stage based on the fact that the Pt particles were only annealed in UHV and given there is no spectroscopic evidence for Pt oxidation. The apparent heights of the Pt particles are typically in the range of 1–2 nm, as seen in the light blue line profile in Figure 4e.

**Figure 4:**
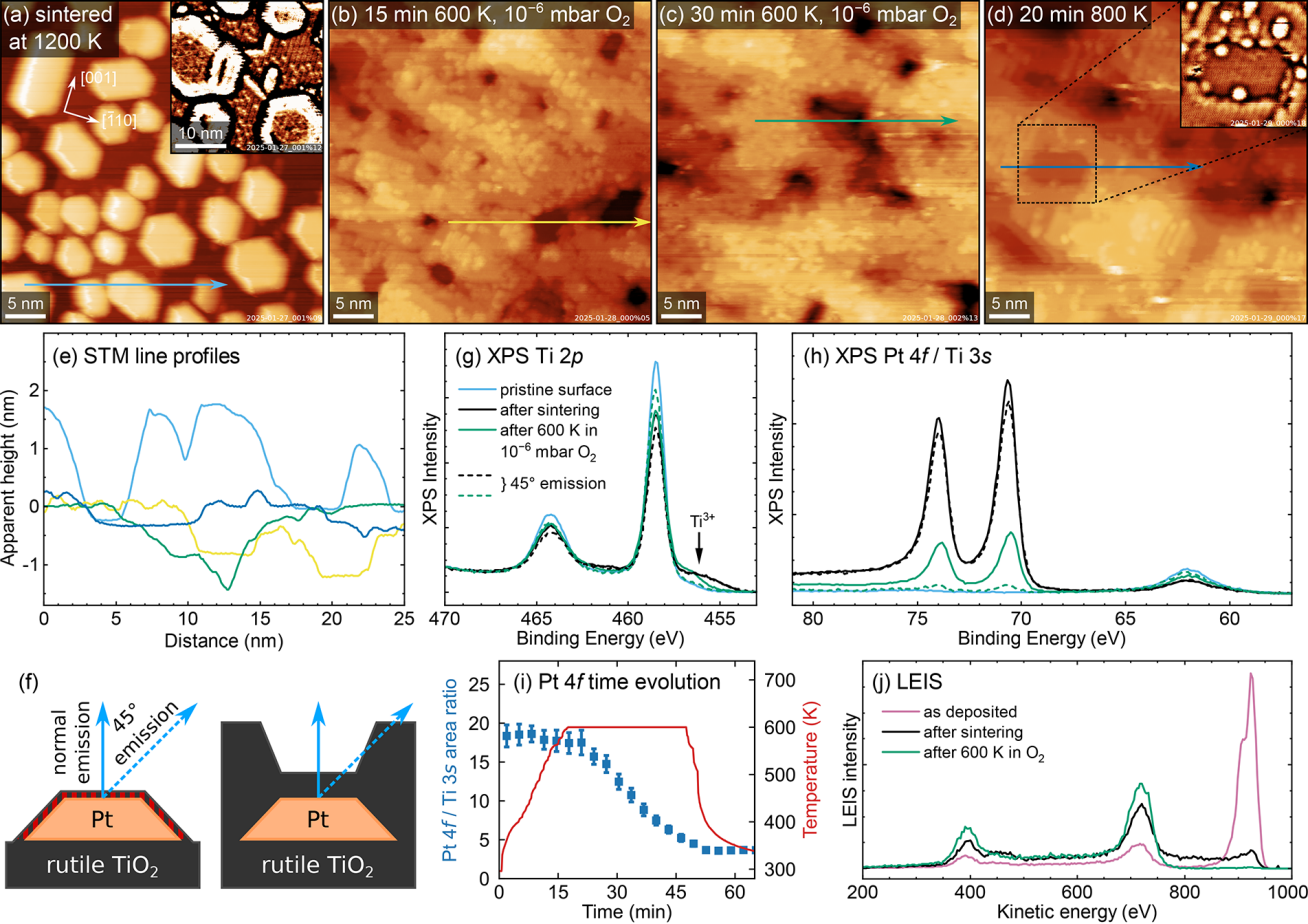
Effect of low-pressure O_2_ exposure on Pt nanoparticles supported on HR-TiO_2_. (a–d) STM images of Pt nanoparticles on HR-TiO_2_ (a) sintered at 1200 K in UHV, then annealed (b) once and (c) twice for 15 min each at 600 K in 10^–6^ mbar O_2_, and (d) postannealed for 20 min at 800 K in UHV. The inset in (a) shows a high-pass-filtered image from the same region, revealing the overlayer structure. The inset in (d) shows a higher-resolution image of the marked area, also high-pass filtered for better visibility. Imaging conditions: RT, UHV, (a) *U*_b_ = 1.5 V, *I*_t_ = 3.3 nA, (b) *U*_b_ = 1.4 V, *I*_t_ = 0.6 nA, (c) *U*_b_ = 1.8 V, *I*_t_ = 1.1 nA, and (d) *U*_b_ = 2.7 V, *I*_t_ = 0.6 nA. (e) Apparent height profiles along the horizontal arrows drawn in (a–d). (f) Schematic illustration of the geometry of (left) encapsulated and (right) buried Pt nanoparticles on HR-TiO_2_(110) and the probing angles for normal and grazing emission XPS. (g,h) XPS spectra (monochromatic Al Kα, normalized to low-binding-energy background) of the Ti 2*p*, Pt 4*f* and Ti 3*s* regions for (blue) pristine TiO_2_(110) before Pt deposition, (black) 7 ML Pt sintered at 1000 K in UHV, and (green) after 30 min at 600 K in 10^–6^ mbar O_2_. Solid lines are at normal emission, dashed lines at 45° emission angle. (i) Area ratios of integrated Pt 4*f* and Ti 3*s* peaks (blue squares) as well as the temperature (red curve) as a function of time, acquired while heating in oxygen under normal emission. (j) LEIS (1025 eV He^+^ ions, 132.5° scattering angle, normalized to full range) acquired in the same experiment as the XPS data in (g–i).

Annealing the sample at 600 K in 10^–6^ mbar O_2_ again leads to the growth of new TiO_2_ layers, as previously seen in Figure 1. STM images after 15 and 30 min O_2_ annealing are shown in Figure 4b,c, respectively. Interestingly, the growth seems to be initially inhibited directly on top of the Pt particles: In an image taken after only 5 min of O_2_ annealing, the encapsulation layer is still clearly visible, as shown in Figure S5, while the surrounding support is already changing significantly. After 15 min, in contrast, no more protruding particles are observed and instead we see pits in the TiO_2_ terraces, with flat regions at the bottom (see Figure 4b and yellow line profile in Figure 4e). In the discussion below, NAP-XPS will allow us to assign these pits to encapsulated particles.

After 30 min of O_2_ annealing, pits are still present, but no flat surfaces could be resolved at their bottom anymore in Figure 4c, suggesting that Pt is becoming fully buried at this point. Postannealing the sample at 800 K for 20 min in UHV also does not recover the particles, as is shown in Figure 4d. While some regions resemble the pits in Figure 4b, closer inspection reveals only the TiO_2_(110)–(1 × 1) structure at their bottom, shown in the inset to Figure 4d. Figure 4f shows a rough schematic of this development: Pt is initially encapsulated by a thin, reduced SMSI layer (dashed red), but becomes deeply buried by new, bulk-stoichiometric TiO_2_ layers when annealed in oxygen. The growth initially seems to be inhibited directly above the particles, but the stoichiometric TiO_2_ layers ultimately also come to cover the platinum, as shown in the right schematic of the final buried state.

As mentioned above, spectroscopy supports this picture: In the XPS data shown in Figure 4h, the Pt 4*f* peak area is significantly diminished after 30 min O_2_ annealing (compare the black curve of the as-sintered nanoparticles with the green curve after O_2_ annealing). This effect is even more pronounced in the more surface-sensitive XPS spectra acquired at 45° emission angle (dashed curves), in line with the schematic picture we draw in Figure 4f. In the Ti 2*p* region shown in Figure 4g, the Ti^3+^ component after sintering Pt (black) is much stronger than for the pristine surface (blue) due to the reduced encapsulating overlayer. This reduction is largely reversed after annealing in O_2_, though the normal-emission spectrum (green, solid) still exhibits more Ti^3+^ than the pristine surface (blue). However, the more surface-sensitive XPS spectra at 45° emission angle (dashed green) reveal that near-surface Ti is already fully reoxidized to the bulk state at this point, suggesting that the additional Ti^3+^ seen in normal emission is located in the vicinity of the buried Pt particles.

Figure 4i shows the time evolution of the Pt 4*f* peak area in proportion to the Ti 3*s* peak area while annealing in oxygen under normal emission, with the corresponding NAP-XPS spectra shown in Figure S6a. Unlike the clusters in Figure 2, the Pt signal from the nanoparticles does not begin to decline until several minutes after reaching 600 K. This can easily be understood by comparison with the STM data: New TiO_2_ layers first need to grow up around the Pt particles, and actual burial of the particles only becomes visible in normal emission geometry after they are already situated in pits. There is no indication of Pt oxidation at any point throughout this measurement series—the Pt 4*f* peak shape remains completely unchanged. However, in the final buried state, an unspecific rising background is observed. This is characteristic for photoelectrons originating at buried species, which have more opportunities to inelastically scatter on the way to the detector,^47^ and is most clearly seen in the Pt 4*d* peak in Figure S7.

Finally, Figure 4j shows LEIS data acquired in the same experiment, sampling different regions of the sample each time to avoid damage from the ion beam in the region where XPS was acquired. The homogeneity of Pt coverage and reduction state was confirmed by XPS throughout. As expected, the spectrum acquired directly after depositing Pt (pink) is dominated by Pt, and the Pt component is drastically diminished after UHV sintering (black) due to SMSI encapsulation. A background-level LEIS signal is still observed in the region leading up to the Pt peak position. This can be assigned as a reionization background due to He scattering at subsurface Pt.^48^ After annealing in oxygen (green line in Figure 4j), this low Pt signal is completely suppressed, again in good agreement with deeper burial of the particles.

### 3.4 Pt Nanoparticles in Near-Ambient Oxygen Pressures

Next, we investigate the same Pt nanoparticles while again increasing the O_2_ pressure. Since we already know from the cluster study that the support growth in 0.1 mbar O_2_ prevents us from obtaining meaningful images by STM, we now focus on spectroscopy. Figure 5 shows the effect of 0.1 mbar O_2_ on Pt nanoparticles supported on both LR-TiO_2_ (Figure 5a–c) and HR-TiO_2_ (Figure 5d–f). As before, 7 ML Pt were deposited on each sample and sintered by annealing at 1000 K in UHV. As we have reported previously,^36^ XPS and LEIS both clearly show that the as-sintered particles are encapsulated on HR-TiO_2_, but not on LR-TiO_2_: The Ti 2*p* peak exhibits a strong Ti^3+^ component on HR-TiO_2_ (Figure 5d) but not on LR-TiO_2_ (Figure 5a), and the LEIS Pt signal decreases only weakly after sintering on LR-TiO_2_ (Figure 5c), but much more strongly on HR-TiO_2_ (Figure 5f).

**Figure 5:**
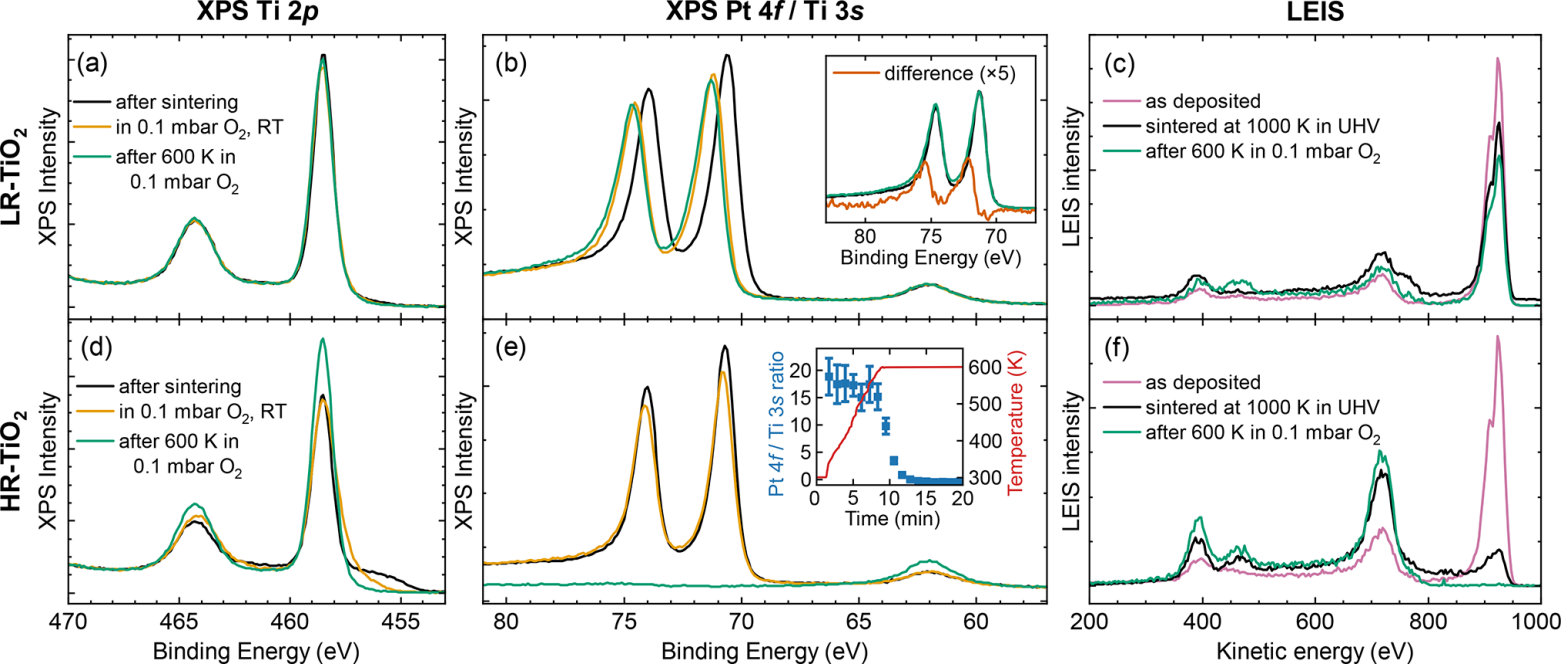
Effect of NAP O_2_ exposure on Pt nanoparticles as a function of TiO_2_ reduction state, with Pt/LR-TiO_2_ in the top row and Pt/HR-TiO_2_ in the bottom row. XPS spectra (monochromatic Al Kα, normalized to low-binding-energy background) of (a,d) the Ti 2*p*, and (b,e) the Pt 4*f* and Ti 3*s* regions at room temperature in UHV after sintering at 600 K (black), at room temperature in 0.1 mbar O_2_ (orange), and at room temperature in UHV after annealing at 600 K in 0.1 mbar O_2_ (green). The inset in (b) compares Pt 4*f* peak shapes before and after O_2_ annealing, with peaks aligned and scaled to match their low-binding-energy edge and the difference spectrum shown in orange. The inset in (e) shows the time evolution of the Pt 4*f* region while heating in oxygen, with (blue) the area ratios of integrated Pt 4*f* and Ti 3*s* peaks and (red) the temperature. (c,f) LEIS (1025 eV He^+^ ions, 132.5° scattering angle, normalized to full range) acquired in the same experiments as the XPS data.

Similar to the effect of 0.1 mbar O_2_ on Pt clusters (Figure 3), the Pt 4*f* peak for nanoparticles on LR-TiO_2_ already shifts substantially relative to the Ti 3*s* peak when exposed to 0.1 mbar O_2_ at room temperature (orange curve in Figure 5b), from 70.6 to 71.2 eV. The effect of annealing at 600 K in O_2_ is less pronounced than for the clusters, but there are still clear indications of Pt oxidation: Directly comparing the peak shapes before and after oxygen treatment, as shown in the inset to Figure 5b, reveals a sharp oxidized component at 72.2 eV. The main Pt 4*f* peak also seems to shift slightly by another 0.1 eV to 71.3 eV. The binding energy difference between the two components is much smaller than for the clusters, which is why we assign this as partial oxidation to Pt^δ+^.

The LEIS data, shown in Figure 5c, confirms that while the Pt nanoparticles are oxidized, they are not becoming encapsulated. The Pt peak after oxidation (green) is only marginally lower than the one directly after sintering, and both are significantly higher than what is typically observed for SMSI-encapsulated Pt (compare to the black curve in Figure 5f). Somewhat unusually, we find that a peak appears at 470 eV kinetic energy, close to the main oxygen peak. We assign this to double-scattering of He^+^ ions from two oxygen atoms, which results in less overall energy loss due to smaller scattering angles.^48,49^ The only chemical species that would yield a peak at a similar position with a single scattering event is fluorine, which was never detected in XPS. Similarly, the Pt peak always exhibits a double line shape, which we also assign to double-scattering.

On HR-TiO_2_, exposure to 0.1 mbar O_2_ at room temperature strongly modifies the appearance of the Ti 2*p* peak in the Ti^3+^ region (compare the black and orange curves in Figure 5d), likely due to interaction with the reduced SMSI overlayer. However, the Pt 4*f* peak (Figure 5e) is affected much less by O_2_ exposure than on LR-TiO_2_, exhibiting only a slight broadening and a tiny shift from 70.7 to 70.8 eV. When heating to 600 K, however, the effect is well in line with that observed for Pt clusters (Figure 2) and for nanoparticles at low O_2_ pressure (Figure 4). Pt quickly becomes deeply buried in oxidatively grown TiO_2_ layers, as seen by the rapid decay of the Pt 4*f* signal, shown in the inset to Figure 5e. The individual NAP-XPS spectra are shown in Figure S6b. After annealing in oxygen at 600 K for 15 min, no Pt signal is detectable any more, and the Ti 2*p* peak appears completely bulk-like. LEIS spectra (Figure 5f) also show the same trends as were already seen at lower pressure (Figure 4j), with no more detectable Pt after oxygen treatment.

### 3.5 Single-Crystalline vs Powder Supports

The experiments we have presented thus far illustrate the key advantage of working with the extremely well-defined single crystalline model systems: even small changes in the support oxidation state, in particle size and oxygen pressure can completely change the oxidation behavior of the Pt/TiO_2_ system, which we can only disentangle in detail by changing one parameter at a time. However, it is equally important to critically evaluate how well the findings from such models can be translated to realistic powder catalysts, where, e.g., the amount of Pt compared to TiO_2_, the oxidation state and the crystallinity might be rather different. In this section, we directly compare the Pt/TiO_2_(110) single crystal model system with a pellet of Pt-loaded P25 powder—the same sample for which electron microscopy studies have been reported in the literature^18,22^ —in NAP-XPS and NEXAFS experiments. Figure S8 shows NEXAFS spectra (Ti L_2,3_ edge) of the powder sample, Pt/LR-TiO_2_(110) and Pt/HR-TiO_2_(110). The curves for the two single crystals differ only slightly, confirming that the bulk crystal structure is the same in both cases. The powder sample also shows similar features but in a different intensity ratio, which could indicate an anatase component in this sample.^50^

NAP-XPS data from the Pt-loaded P25 powder are shown in Figure 6a. Unlike for the single crystal samples, all XPS data had to be acquired at elevated temperatures in gas atmosphere due to poor conductivity of the pressed pellet at RT in UHV. The top (black) spectrum in Figure 6a was acquired in 1 mbar H_2_ at 870 K to mimic the reductive UHV annealing step while avoiding charging of the powder sample. Pt appears fully metallic, with a similar peak shape as observed for nanoparticles on the single-crystalline samples. The peak shape of this metallic component was kept fixed for all further fits.

**Figure 6:**
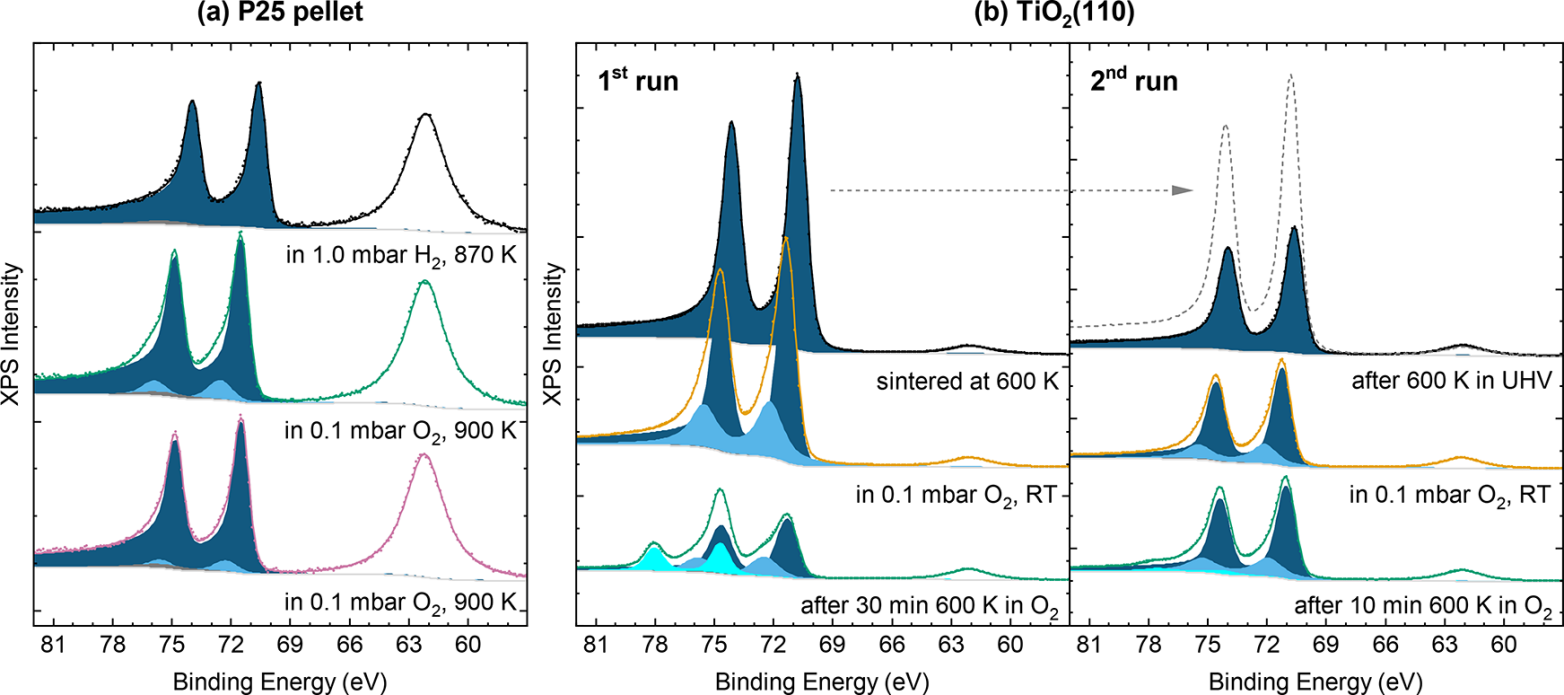
NAP-XPS spectra (*h*ν = 650 eV, normalized to Ti 3*s* peak) showing the Pt 4*f* and Ti 3*s* region (a) for a pellet of Pt/P25 powder catalyst as used in ref 18 and (b) for Pt on HR-TiO_2_. In (a), all spectra were acquired at elevated temperatures on the powder catalyst pellet due to poor conductivity at room temperature. The bottom (pink) spectrum was acquired under the same conditions as the middle (green) one, after cooling to room temperature, then heating back up to 900 K in oxygen. In (b), Pt was deposited on an HR-TiO_2_(110) single crystal, sintered at 600 K (top left, black), then exposed to 0.1 mbar O_2_ at room temperature (middle, orange) and heated to 600 K in O_2_. The bottom (green) spectra were acquired after returning to room temperature and UHV. The right panel in (b) shows a second run of O_2_ annealing, acquired after annealing the same sample at 600 K in UHV again; the dashed gray line is the original as-sintered spectrum (top left, black) for comparison.

After cooling to room temperature, replacing the H_2_ gas with 0.1 mbar O_2_ and heating to 900 K (green curve in Figure 6a), a clear oxidized Pt 4*f* component appears at 72.6 eV. The main metallic peak shifts from 70.6 to 71.4 eV, placing it very close to the 71.3 eV seen for Pt nanoparticles on LR-TiO_2_(110) (Figure 5b). Interestingly, this initial oxidation is not fully stable: After cooling the sample back to room temperature, then heating again to nominally the same conditions as before, platinum appears to be less oxidized (pink curve in Figure 6a). While the main peak remains at 71.4 eV, the oxidized component is less pronounced, and now appears at 72.3 eV. No further modification of this end state was observed, and we conclude that the original stronger oxidation (green curve) is due to a metastable configuration, which we can also mimic in our model system, as shown below in Figure 6b. It is worth noting that in the final configuration, the distance between the main peak and the oxidized component is 0.9 eV, in excellent agreement with that observed for Pt nanoparticles on LR-TiO_2_ (Figure 5b).

### 3.6 Effect of Particle Crystallinity

For a direct comparison in the same NAP-XPS instrument as used for Pt-loaded P25 powder, Pt was again deposited onto HR-TiO_2_. Based on SESSA simulations, we estimate the coverage to be about 3 ML. In this experiment, the Pt/HR-TiO_2_ sample was sintered at only 600 K, leading to a smaller average particle size than in the nanoparticle experiments presented above. As a result, the oxidation behavior in 0.1 mbar O_2_ at 600 K shown in Figure 6b also lies in between that observed for clusters (Figure 2) and for nanoparticles (Figure 5): Despite the high coverage, Pt becomes strongly oxidized, which was not observed on well-crystallized nanoparticles. Interestingly, the Pt signal is also not completely suppressed even after 30 min in O_2_ (green curve in first run of Figure 6b).

It becomes clear that this is due to the less crystalline nature of the original state when following up with another cycle of the same experiment, on the same sample, shown in the right panel of Figure 6b. Here, the top (black) spectrum was acquired after once more annealing the sample at 600 K in UHV, directly subsequent to the experiment shown in the left panel of Figure 6b. This results in a fully metallic Pt peak, though significantly lower than the initial as-sintered state (shown again as a dashed gray line for comparison). Repeating the exposure to oxygen and annealing results in much less oxidation than in the first O_2_ annealing step, presumably due to the particles becoming more well-sintered and crystalline during the first round of oxidation and reduction. This metastable oxide formation is comparable in the HR-TiO_2_ and the powder sample. In all three samples—Pt/P25, Pt/HR-TiO_2_ and Pt/LR-TiO_2_(110)—the NEXAFS spectra (shown in Figure S9) remain largely unchanged throughout the experiments, indicating that there is no major bulk restructuring. That being said, after finishing the entire round of experiments, the 0.5 mm thick TiO_2_ crystal had lost most of its original oblique-black color, indicating bulk titania reoxidation.

## 4 Discussion

Two main themes emerge from all the results presented above: First, the degree to which platinum can be oxidized varies with particle size and oxygen pressure. Second, we describe a new deactivation mechanism, namely the oxidative deep burial of platinum in the support, which is not limited by direct interactions with the particles, in contrast to the shallow, self-limiting encapsulation in the nonclassical SMSI.^18,22^ This burial is surprisingly fast even at only 600 K, indicating a high rate of bulk diffusion for Ti interstitials. The dependence on the availability of Ti interstitials obviously limits the comparability of powder TiO_2_ to single crystals, which have a much larger bulk reservoir.

### 4.1 Size-Dependence of Pt Oxidation

We find substantial differences when we directly compare the oxidation of Pt nanoparticles and subnanometer clusters on LR-TiO_2_ upon exposure to 0.1 mbar O_2_. Since the bulk of the larger Pt nanoparticles remains metallic, a much larger fraction of Pt atoms is oxidized for clusters (Figure 3) than for nanoparticles (Figure 5b). In addition, there is also a clear qualitative difference in the degree of oxidation: the peak-to-peak separation between the least and most oxidized components is significantly larger for clusters (2.4 eV) than for nanoparticles (0.9 eV), with the most oxidized component for clusters appearing at 74.8 eV. While peaks are typically shifted to higher binding energy for small clusters due to final-state effects,^51–54^ making it difficult to directly assign oxidation states, the peak-to-peak separation still clearly indicates the formation of a highly oxidized Pt species, which we assign to Pt^2+^. The nanoparticles, in contrast, are only oxidized to an intermediate Pt^δ+^ state: A similar oxidized peak at +0.9 eV with respect to Pt^0^ was observed in a previous NAP-XPS study on bulk Pt(111) after annealing in 0.5 Torr (0.67 mbar) O_2_, and was interpreted as a surface oxide.^55^ Bulk-like platinum oxide with a highly oxidized Pt 4*f* component, comparable to that seen here for the clusters, was only formed when annealing Pt(111) to 720 K in 5 Torr (6.67 mbar) O_2_.^55^ Pt can likely achieve a higher oxidation state in clusters than in nanoparticles because the already low-coordinated surface atoms can restructure relatively easily into highly O-coordinated motifs. This is in good agreement with previous studies of small Pt clusters on ceria, where theory predicts the formation of PtO_*x*_ clusters in oxygen-rich conditions, with all Pt–Pt bonds replaced by Pt–O–Pt.^27,30,56^ Interestingly, cluster oxidation appears to be facile even at room temperature, with initial Pt oxidation seen in XPS on both LR-TiO_2_ and HR-TiO_2_ when Pt clusters are exposed to 0.1 mbar O_2_ (orange curves in Figure 3b,d).

The hypothesis that the degree of Pt oxidation is largely dependent on its initial coordination is further supported by the behavior of less well-crystallized Pt nanoparticles, sintered at a lower temperature (Figure 6b). Here, strongly oxidized components are observed even for very high Pt loadings, more comparable to Pt clusters than to fully crystallized nanoparticles, sintered at 1000 K. We also found a similar behavior with highly oxidized components in previous work, where submonolayer Pt coverages were sintered at 800 K.^40^ We interpret this observation as a stronger interaction of oxygen with under-coordinated Pt, which would be present both in clusters and in not yet fully crystallized nanoparticles or films. Cycling oxygen and UHV annealing ultimately reduces the degree to which particles can be oxidized (Figure 6b), likely because Pt sinters to larger and more crystalline particles in the process. Direct comparison to large P25-supported Pt nanoparticles (Figure 6a) also supports this view, as these behave much like the well-crystallized Pt nanoparticles on LR-TiO_2_ (Figure 5b) and HR-TiO_2_ after long O_2_ annealing (Figure 6b), and do not exhibit any highly oxidized components.

Finally, we also consider the formation of a Ti–Pt–O mixed oxide when small Pt clusters are annealed in NAP O_2_. In nanoparticles, Ti–Pt alloying has been reported under reducing conditions,^18,57^ but oxidizing atmospheres typically lead to resegregation of TiO_*x*_ in a “non-classical SMSI” effect.^18^ This is expected based on thermodynamic considerations: The standard enthalpy of formation Δ_f_H^0^ of rutile TiO_2_ (939 kJ/mol)^58^ is about three times higher per Ti atom than that of bulk TiPt_3_ (298 kJ/mol),^59^ and we are not aware of any reported Ti–Pt–O bulk oxide phase. Overall, while we cannot fully exclude the possibility of a (transient) mixed phase, we conclude that the oxidation of Pt to PtO_*x*_ clusters is more likely to proceed without Ti–Pt mixing.

### 4.2 Oxidative Deactivation of Pt on HR-TiO_2_

A previous study investigated the CO oxidation activity of Pt clusters on LR-TiO_2_ and HR-TiO_2_ at low pressures and came to the conclusion that HR-TiO_2_ is less active by 2 orders of magnitude because Pt competes for oxygen with the support.^60^ Clearly, our results show that Pt on (single-crystalline) HR-TiO_2_ is a poor oxidation catalyst, due to oxidative TiO_2_ layer growth leading to rapid burial of Pt particles at all pressures and sizes.

It is plausible that this buried state is not the thermodynamic minimum, but rather a result of kinetic limitations. Notably, TiO_2_ does not appear to preferentially overgrow the Pt nanoparticles. At low oxygen pressure, the reduced SMSI overlayer remains intact even as the surrounding TiO_2_ becomes oxidized (Figure S5). This suggests that replacing or overgrowing the encapsulation layer with stoichiometric TiO_2_ may be energetically costly because of interface strain, which the thin overlayer can more easily compensate than (1 × 1)-periodic, bulk-like TiO_2_. Only once the particles are situated at the bottom of newly grown pits, which also incur an energy cost, do they become overgrown by bulk-like TiO_2_ layers. From an energetic point of view, it is likely that placing the particle on top of the TiO_2_ crystal, rather than as a subsurface inclusion, would be preferred.

Interestingly, the only instance in which Pt on HR-TiO_2_ is not fully buried after annealing in 0.1 mbar O_2_ is the partially sintered high Pt coverage, shown in Figure 6b. As discussed above, Pt in this configuration appears more susceptible to oxidation, indicating that an encapsulation layer is absent or at least incomplete. We can speculate, then, that more oxidized, larger Pt particles can better resist the burial. This is in agreement with our previous study where size-selected Pt_10_ clusters were deposited intact.^40^ The enhanced sintering of Pt clusters in 0.1 mbar O_2_ (Figure 2) implies faster diffusion rates, which may allow at least some fraction of Pt to “float” on the growing TiO_2_ layers. Such a floating effect would be made significantly more difficult by a complete SMSI encapsulation layer. We conclude that the buried configuration of Pt particles is likely metastable, and arises only because oxidative layer growth on the bare TiO_2_ is facile compared to Pt particle diffusion.

More generally, we speculate that the same mechanisms as described here for Pt on TiO_2_ would also lead to burial of metal particles on other reducible oxide supports which exhibit SMSI encapsulation and cation-dominated bulk defect chemistry, such as magnetite (Fe_3_O_4_)^20,61^ as well as other spinel and rutile oxides. It would be interesting to contrast this to substrates where bulk reduction is primarily defined by oxygen vacancy defects, such as ceria. Presumably, no oxidative burial would be observed there, as substrate reoxidation would not involve the growth of new layers.

### 4.3 TiO_2_ Reoxidation Rate

The extremely fast burial of Pt in near-ambient pressure oxygen, to the point where it is not detectable by XPS any more, is somewhat surprising. The NIST Electron Effective-Attenuation-Length Database^62^ predicts an inelastic mean free path (IMFP) for rutile of 2.8 nm at the kinetic energy of the Pt 4*f*_7/2_ peak (1415 eV with Al Kα excitation). Attenuating the Pt signal below 5% (1%) of its initial value would take 7.3 nm (11.2 nm) of added rutile thickness,^62^ requiring a substantial number of Ti atoms to be drawn from the bulk. This is accomplished in 0.1 mbar O_2_ within 5 min of reaching 600 K on HR-TiO_2_ (see inset to Figure 5d).

The depth from which Ti_int_ can reach the surface while annealing depends on the Ti_int_ diffusion barriers one assumes, but may be surprisingly large: With a 0.5 eV barrier,^63–65^ a simple random-walk model of noninteracting Ti_int_, discussed in more detail elsewhere,^36^ yields a characteristic diffusion length of 0.14 mm for only 5 min at 600 K. A more thorough calculation for the number of Ti_int_ reaching the surface within a given time is given in the Supporting Information. We calculate that up to 22.7 nm of new TiO_2_ layers may be grown in 5 min at 600 K if every Ti_int_ that randomly diffuses to the surface is oxidized. Conversely, we can use the same model to find an upper limit for the Ti_int_ bulk diffusion barrier, *E*_*B*_ = 0.62 eV, above which the observed Pt attenuation could not be explained (see Supporting Information for details).

This diffusion model does not account for any additional barriers in rearranging the newly oxidized TiO_2_ moieties at the surface into new bulk-like TiO_2_ terraces,^36–38^ or a possible higher barrier for diffusion from the subsurface to the surface.^66^ It seems likely that these would be rate-limiting for surface layer growth, at least initially. However, the estimates we have made are extremely conservative, and the calculated maximum bulk diffusion barrier of 0.62 eV is still relatively close to the previously reported 0.5 eV.^63–65^ The possibility thus still remains that bulk Ti_int_ transport limits the layer growth from the beginning even on HR-TiO_2_, and it should quickly become dominant for more stoichiometric samples. Even with a barrier on the order of 0.6 eV, however, diffusion and bulk reoxidation clearly proceed much faster than intuition might suggest. This has severe implications for the comparison between bulk rutile crystals and TiO_2_ powder samples.

In a previous study, some of the present authors had observed that submonolayer Pt coverages were buried less quickly at high than at intermediate oxygen pressure.^40^ The stoichiometry of the TiO_2_ samples was not strictly controlled in that work, but based on preparation and sample color, we estimate that they were slightly more reduced than the LR-TiO_2_ used here, and much less reduced than HR-TiO_2_. The rate of burial was also compared as a function of oxygen dose, rather than of time. With such a description, the present data would similarly suggest a “faster” burial at low pressure: The Pt signal decreases about an order of magnitude more slowly in 0.1 mbar O_2_ (Figure 5) than in 10^–6^ mbar O_2_ (Figure 4), but this is easily compensated by the 5 orders of magnitude pressure difference. That being said, the availability of gas-phase oxygen may no longer be rate-limiting to the reoxidation process at 600 K in 0.1 mbar O_2_, as implied when referring to the oxygen dose: The availability of Ti_int_, together with possible barriers for surface reorganization, are sufficient to limit the growth rate to the experimentally observed value. We therefore conclude that although reoxidation rates depend linearly on the oxygen pressure in the 10^–6^–10^–7^ mbar range,^37^ this is not the case at elevated pressure, where Ti_int_ availability instead becomes rate-limiting.

It is interesting to frame this in terms of a “pressure gap”: When the system is in a diffusion-limited regime (i.e., lower Ti_int_ flux than O_2_ impingement at the surface), the TiO_2_ growth rate becomes largely insensitive to pressure. However, the crossover from pressure-limited to diffusion-limited kinetics is exactly what causes a pressure gap to appear: The linear relationship between oxygen pressure and TiO_2_ growth rate, identified by varying *p*_O2_ in the UHV-compatible range,^37^ cannot be extrapolated indefinitely to higher pressures.

### 4.4 Validity of Model Systems and Lessons for Powder Catalysts

The deep burial of Pt through oxidative layer growth is clearly not directly transferrable to Pt nanoparticles supported on powder TiO_2_, simply because there is no comparably large bulk reservoir of Ti^3+^ interstitials. Highly reduced rutile TiO_2–*x*_ still only contains a relatively small amount of excess Ti, with *x* on the order of 4 × 10^–4^.^32^ This implies that the volume of a TiO_2_ particle can only increase by about 0.04% when moving from highly reduced to a fully oxidized stoichiometry. Due to the extremely fast Ti_int_ diffusion and the large volume of TiO_2_, this can still amount to hundreds of nanometers on millimeter-sized single crystals. In contrast, TiO_2_ powder samples have a much smaller TiO_2_ to Pt volume ratio and will thus rapidly deplete their bulk of excess Ti_int_, limiting the growth.

The flipside of this difference in bulk reservoir is that we can also expect powders to rapidly change their bulk oxidation state in reducing or oxidizing environments—unlike single crystals. For example, if we assume TiO_2_ particles with a radius of 100 nm, then 0.04% growth amounts to only 0.2 Å (!), and would be finished in seconds at 600 K. Surface reaction barriers may limit this rate somewhat. Nonetheless, on a typical experimental time scale, we would expect that there is essentially no delay between changing the chemical potential of the gas environment, and the stoichiometry of a powder TiO_2_ sample following suit. This has important implications for the electronic interactions of metal particles and their supports, but also for the study of SMSI encapsulation and de-encapsulation: If the TiO_2_ stoichiometry is strongly coupled to the gas atmosphere, the direct effects of the gas atmosphere on supported metal particles are impossible to disentangle from the effects of changing metal–support interaction.

Single crystal model systems can alleviate this issue. Due to the much larger bulk reservoir, the support stoichiometry can be kept approximately constant throughout a typical experiment, while the gas environment can be switched in a matter of minutes. This allows a distinction between how a change in the gas atmosphere (i) directly affects the Pt particles themselves, and (ii) indirectly affects the particles by modifying the supporting oxide. For example, an oxidizing atmosphere does not appear to modify the reduced encapsulation layer on HR-TiO_2_ (Figure S5), and even extended UHV annealing at 1100 K does not lead to encapsulation of Pt particles on LR-TiO_2_.^36^ In both cases, we can conclude that the interaction of the particles with an oxidizing or reducing environment is not enough in itself: It takes a modification of the TiO_2_ support to create or remove an overlayer.

We conclude that model studies on single crystal supports can be extremely helpful for mechanistic understanding, but care must be taken to control all relevant variables. When using single crystals as a stand-in for powder samples, a good rule of thumb is that the single crystal stoichiometry should mirror the one expected for the powder under the given conditions. Here, we find that under oxidizing conditions, Pt nanoparticles on LR-TiO_2_ behave very similarly to particles on P25, which fits with the assumption that the powder is fully oxidized in 0.1 mbar O_2_. In contrast, HR-TiO_2_ is the better model for powder TiO_2_ under reducing atmospheres. Precisely controlling the stoichiometry of oxide single crystals,^36^ rather than allowing it to drift over many sputtering and annealing cycles, is thus essential in order to get reproducible and interpretable results. We predict that this holds not only for TiO_2_, but for all reducible oxide supports, in particular when they exhibit rapid ion transport.

## 5 Conclusions

We have presented a systematic study of Pt oxidation on rutile TiO_2_ where we control the particle size, oxygen pressure, and support stoichiometry. On near-stoichiometric TiO_2_, both Pt nanoparticles and subnanometer clusters become oxidized in 0.1 mbar O_2_ at 600 K. Clusters are oxidized even at room temperature, and their oxidation at 600 K is much more pronounced than that of nanoparticles, both in terms of the fraction of oxidized Pt and of the highest oxidation state. In general, the degree of Pt oxidation is largely dependent on its coordination or crystallinity.

On reduced TiO_2_, we have described the rapid burial of Pt particles by the TiO_2_ support through oxidative layer growth. The high rate of support reoxidation implies rapid Ti interstitial diffusion. The significantly lower number of Ti_int_ available in nanoscale TiO_2_ powder grains thus suggests that TiO_2_ bulk stoichiometry can be modified within minutes for powder samples. Model systems must thus be prepared with well-defined stoichiometries to fit the state of a real catalyst under the respective atmosphere.

## Supplementary Material



## Data Availability

The data is available on: 10.5281/zenodo.17233126

## Notes

The following files are available free of charge. Description of SESSA simulations, discussion of bulk diffusion lengths during TiO_2_ reoxidation, supplementary figures (PDF)

## ⊥

Institute of Applied Physics, TU Wien, 1040 Vienna, Austria

## Abbreviations

HR-TiO_2_: highly reduced TiO_2_
LEIS: low-energy ion scattering
LR-TiO_2_: low-reduced TiO_2_
ML: monolayer
NAP: near-ambient pressure
NEXAFS: near-edge X-ray absorption fine structure
RT: room temperature
SMSI: strong metal–support interaction
STM: scanning tunneling microscopy
UHV: ultrahigh vacuum
XPS: X-ray photoelectron spectroscopy.

## References

[1] Engel, T. ; Ertl, G. Elementary Steps in the Catalytic Oxidation of Carbon Monoxide on Platinum Metals. In Adv. Catal.; Eley, D. D. , Pines, H. , Weez, P. B. , Eds.; Academic Press, 1979; Vol. 28, pp 1–78. 10.1016/S0360-0564(08)60133-9.

[2] Rodriguez J. A., Goodman D. W. (1991). High-pressure catalytic
reactions over single-crystal
metal surfaces. Surf. Sci. Rep..

[3] Van
Spronsen M. A., Frenken J. W. M., Groot I. M. N. (2017). Surface science
under reaction conditions: CO oxidation on Pt and Pd model catalysts. Chem. Soc. Rev..

[4] Schlögl R. (2015). Heterogeneous
Catalysis. Angew. Chem., Int. Ed..

[5] Tao F., Salmeron M. (2024). Surface restructuring
and predictive design of heterogeneous
catalysts. Science.

[6] Xu L., Papanikolaou K. G., Lechner B. A. J., Je L., Somorjai G. A., Salmeron M., Mavrikakis M. (2023). Formation
of active sites on transition
metals through reaction-driven migration of surface atoms. Science.

[7] Kaiser S., Maleki F., Zhang K., Harbich W., Heiz U., Tosoni S., Lechner B. A. J., Pacchioni G., Esch F. (2021). Cluster Catalysis with Lattice Oxygen: Tracing Oxygen Transport from
a Magnetite (001) Support onto Small Pt Clusters. ACS Catal..

[8] Kaiser S., Plansky J., Krinninger M., Shavorskiy A., Zhu S., Heiz U., Esch F., Lechner B. A. J. (2023). Does Cluster
Encapsulation Inhibit Sintering? Stabilization of Size-Selected Pt
Clusters on Fe_3_O_4_(001) by SMSI. ACS Catal..

[9] Lykhach Y., Kozlov S. M., Skála T., Tovt A., Stetsovych V., Tsud N., Dvořák F., Johánek V., Neitzel A., Mysliveček J., Fabris S., Matolín V., Neyman K. M., Libuda J. (2016). Counting electrons
on supported nanoparticles. Nat. Mater..

[10] Campbell C. T. (2012). Electronic
perturbations. Nat. Chem..

[11] Tauster S. J. (1987). Strong
metal-support interactions. Acc. Chem. Res..

[12] Tauster S. J., Fung S. C., Garten R. L. (1978). Strong
metal-support interactions.
Group 8 noble metals supported on titanium dioxide. J. Am. Chem. Soc..

[13] Pesty F., Steinrück H.-P., Madey T. E. (1995). Thermal stability of Pt films on
TiO_2_(110): evidence for encapsulation. Surf. Sci..

[14] Dulub O., Hebenstreit W., Diebold U. (2000). Imaging Cluster Surfaces with Atomic
Resolution: The Strong Metal-Support Interaction State of Pt Supported
on TiO_2_(110). Phys. Rev. Lett..

[15] Bennett R. A., Pang C. L., Perkins N., Smith R. D., Morrall P., Kvon R. I., Bowker M. (2002). Surface Structures
in the SMSI State;
Pd on (1 × 2) Reconstructed TiO_2_(110). J. Phys. Chem. B.

[16] Bowker M., Stone P., Morrall P., Smith R., Bennett R., Perkins N., Kvon R., Pang C., Fourre E., Hall M. (2005). Model catalyst studies of the strong metal–support interaction:
Surface structure identified by STM on Pd nanoparticles on TiO_2_(110). J. Catal..

[17] Fu Q., Wagner T., Olliges S., Carstanjen H.-D. (2005). Metal–Oxide
Interfacial Reactions: Encapsulation of Pd on TiO_2_ (110). J. Phys. Chem. B.

[18] Beck A., Huang X., Artiglia L., Zabilskiy M., Wang X., Rzepka P., Palagin D., Willinger M.-G., van Bokhoven J. A. (2020). The dynamics of overlayer formation on catalyst nanoparticles
and strong metal-support interaction. Nat. Commun..

[19] Tauster S. J., Fung S. C. (1978). Strong metal-support
interactions: Occurrence among
the binary oxides of groups IIA–VB. J.
Catal..

[20] Qin Z. H., Lewandowski M., Sun Y. N., Shaikhutdinov S., Freund H. J. (2008). Encapsulation of
Pt Nanoparticles as a Result of Strong
Metal–Support Interaction with Fe_3_O_4_(111). J. Phys. Chem. C.

[21] Xu M., Peng M., Tang H., Zhou W., Qiao B., Ma D. (2024). Renaissance of Strong Metal–Support Interactions. J. Am. Chem. Soc..

[22] Frey H., Beck A., Huang X., van Bokhoven J. A., Willinger M. G. (2022). Dynamic interplay between metal nanoparticles and oxide
support under redox conditions. Science.

[23] Matsubu J. C., Zhang S., Derita L., Marinkovic N. S., Chen J. G., Graham G. W., Pan X., Christopher P. (2017). Adsorbate-mediated
strong metal–support interactions in oxide-supported Rh catalysts. Nat. Chem..

[24] Monai M., Jenkinson K., Melcherts A. E. M., Louwen J. N., Irmak E. A., Van Aert S., Altantzis T., Vogt C., van der
Stam W., Duchoň T., Šmíd B., Groeneveld E., Berben P., Bals S., Weckhuysen B. M. (2023). Restructuring
of titanium oxide overlayers over nickel nanoparticles during catalysis. Science.

[25] Herzing A. A., Kiely C. J., Carley A. F., Landon P., Hutchings G. J. (2008). Identification
of Active Gold Nanoclusters on Iron Oxide Supports for CO Oxidation. Science.

[26] Crampton A. S., Rötzer M. D., Landman U., Heiz U. (2017). Can Support Acidity
Predict Sub-Nanometer Catalyst Activity Trends?. ACS Catal..

[27] Slavinskaya E. M., Stadnichenko A. I., Quinlivan Domínguez J. E., Stonkus O. A., Vorokhta M., Šmíd B., Castro-Latorre P., Bruix A., Neyman K. M., Boronin A. I. (2023). States of Pt/CeO_2_ catalysts for CO oxidation below room temperature. J. Catal..

[28] Gao F., McClure S. M., Cai Y., Gath K. K., Wang Y., Chen M. S., Guo Q. L., Goodman D. W. (2009). CO oxidation trends
on Pt-group metals from ultrahigh vacuum to near atmospheric pressures:
A combined in situ PM-IRAS and reaction kinetics study. Surf. Sci..

[29] Eads C. N., Wang W., Küst U., Prumbs J., Temperton R. H., Scardamaglia M., Schnadt J., Knudsen J., Shavorskiy A. (2025). Resolving
active species during the carbon monoxide oxidation over Pt(111) on
the microsecond timescale. Nat. Commun..

[30] Wang H., Liu J.-X., Allard L. F., Lee S., Liu J., Li H., Wang J., Wang J., Oh S. H., Li W., Flytzani-Stephanopoulos M., Shen M., Goldsmith B. R., Yang M. (2019). Surpassing the single-atom
catalytic activity limit through paired
Pt-O-Pt ensemble built from isolated Pt_1_ atoms. Nat. Commun..

[31] Henderson M. A. (1999). A surface
perspective on self-diffusion in rutile TiO_2_. Surf. Sci..

[32] Aono M., Hasiguti R. R. (1993). Interaction and ordering of lattice
defects in oxygen-deficient
rutile TiO_2–x_. Phys. Rev.
B.

[33] Onishi H., Fukui K. i., Iwasawa Y. (1995). Atomic-Scale
Surface Structures of
TiO_2_(110) Determined by Scanning Tunneling Microscopy:
A New Surface-Limited Phase of Titanium Oxide. Bull. Chem. Soc. Jpn..

[34] Li M., Hebenstreit W., Gross L., Diebold U., Henderson M. A., Jennison D. R., Schultz P. A., Sears M. P. (1999). Oxygen-induced restructuring
of the TiO_2_(110) surface: a comprehensive study. Surf. Sci..

[35] Li M., Hebenstreit W., Diebold U. (2000). Morphology change of oxygen-restructured
TiO_2_(110) surfaces by UHV annealing: Formation of a low-temperature
(1 × 2) structure. Phys. Rev. B.

[36] Kraushofer F., Krinninger M., Kaiser S., Reich J., Jarosz A., Füchsl M., Anand G., Esch F., Lechner B. A. J. (2024). The
influence of bulk stoichiometry on near-ambient pressure reactivity
of bare and Pt-loaded rutile TiO_2_(110). Nanoscale.

[37] Bowker M., Bennett R. A. (2009). The role of Ti^3+^ interstitials in TiO_2_(110) reduction and oxidation. J. Phys.:
Condens. Matter.

[38] Bennett R. A., Stone P., Price N. J., Bowker M. (1999). Two (1 × 2) Reconstructions
of TiO_2_(110): Surface Rearrangement and Reactivity Studied
Using Elevated Temperature Scanning Tunneling Microscopy. Phys. Rev. Lett..

[39] Bennett R. A., Stone P., Bowker M. (1999). Pd nanoparticle enhanced
re-oxidation
of non-stoichiometric TiO_2_: STM imaging of spillover and
a new form of SMSI. Catal. Lett..

[40] Petzoldt P., Eder M., Mackewicz S., Blum M., Kratky T., Günther S., Tschurl M., Heiz U., Lechner B. A. J. (2022). Tuning
Strong Metal–Support Interaction Kinetics on Pt-Loaded TiO_2_(110) by Choosing the Pressure: A Combined Ultrahigh Vacuum/Near-Ambient
Pressure XPS Study. J. Phys. Chem. C.

[41] Crumlin E. J., Liu Z., Bluhm H., Yang W., Guo J., Hussain Z. (2015). X-ray spectroscopy
of energy materials under in situ/operando conditions. J. Electron Spectrosc. Relat. Phenom..

[42] Grass M. E., Karlsson P. G., Aksoy F., Lundqvist M., Wannberg B., Mun B. S., Hussain Z., Liu Z. (2010). New ambient
pressure photoemission endstation at Advanced Light Source beamline
9.3.2. Rev. Sci. Instrum..

[43] Wendt S., Sprunger P. T., Lira E., Madsen G. K. H., Li Z., Hansen J. Ø., Matthiesen J., Blekinge-Rasmussen A., Lægsgaard E., Hammer B., Besenbacher F. (2008). The Role of
Interstitial Sites in the Ti*3d* Defect State in the
Band Gap of Titania. Science.

[44] Smekal W., Werner W. S. M., Powell C. J. (2005). Simulation of electron
spectra for
surface analysis (SESSA): a novel software tool for quantitative Auger-electron
spectroscopy and X-ray photoelectron spectroscopy. Surf. Interface Anal..

[45] Werner, W. ; Smekal, W. ; Powell, C. J. , NIST Database for the Simulation of Electron Spectra for Surface Analysis (SESSA), SRD 100, Version 2.1. 2017.

[46] Van
Spronsen M. A., Frenken J. W. M., Groot I. M. N. (2017). Observing the
oxidation of platinum. Nat. Commun..

[47] Tougaard S. (2018). Improved XPS
analysis by visual inspection of the survey spectrum. Surf. Interface Anal..

[48] Průša S., Linford M. R., Vaníčková E., Bábík P., Pinder J. W., Šikola T., Brongersma H. H. (2024). A practical guide to interpreting low energy ion scattering
(LEIS) spectra. Appl. Surf. Sci..

[49] Niehus H., Heiland W., Taglauer E. (1993). Low-energy ion scattering
at surfaces. Surf. Sci. Rep..

[50] Ruus R., Kikas A., Saar A., Ausmees A., Nõmmiste E., Aarik J., Aidla A., Uustare T., Martinson I. (1997). Ti 2*p* and O 1*s* X-ray absorption of TiO_2_ polymorphs. Solid State Commun..

[51] Wertheim G. K., Dicenzo S. B., Youngquist S. E. (1983). Unit Charge
on Supported Gold Clusters
in Photoemission Final State. Phys. Rev. Lett..

[52] Tamura K., Kudo M., Owari M., Nihei Y. (1986). X-Ray Photoelectron
Diffraction (XPED) Studies of Platinum on Titanium Dioxide (110) Surface. Chem. Lett..

[53] Steinrück H.-P., Pesty F., Zhang L., Madey T. E. (1995). Ultrathin films
of Pt on TiO_2_(110): Growth and chemisorption-induced surfactant
effects. Phys. Rev. B.

[54] Perco D., Pozzo M., Berti A., Loi F., Lacovig P., Lizzit S., Kartouzian A., Heiz U., Alfè D., Baraldi A. (2025). Limitations in Determining
Oxidation States in Condensed
Matter at the Subnanometric Scale. J. Am. Chem.
Soc..

[55] Miller D. J., Öberg H., Kaya S., Sanchez Casalongue H., Friebel D., Anniyev T., Ogasawara H., Bluhm H., Pettersson L. G. M., Nilsson A. (2011). Oxidation of Pt(111)
under Near-Ambient Conditions. Phys. Rev. Lett..

[56] Quinlivan
Domínguez J. E., Neyman K. M., Bruix A. (2022). Stability of oxidized
states of freestanding and ceria-supported PtO_x_ particles. J. Chem. Phys..

[57] Beard B. C., Ross P. N. (1986). Platinum-titanium
alloy formation from high-temperature
reduction of a titania-impregnated platinum catalyst: implications
for strong metal-support interaction. J. Phys.
Chem..

[58] Chase, M. W. NIST-JANAF Thermochemical Tables. 4 ed.; National Institute of Standards and Technology: Gaithersburg, MD, 1998.

[59] Meschter P. J., Worrell W. L. (1976). An investigation of high-temperature thermodynamic
properties in the Pt-Ti system. Metall. Trans.
A.

[60] Bonanni S., Aït-Mansour K., Harbich W., Brune H. (2012). Effect of the TiO_2_ Reduction
State on the Catalytic CO Oxidation on Deposited
Size-Selected Pt Clusters. J. Am. Chem. Soc..

[61] Tober S., Creutzburg M., Arndt B., Krausert K., Mattauch S., Koutsioubas A., Pütter S., Mohd A. S., Volgger L., Hutter H., Noei H., Vonk V., Lott D., Stierle A. (2020). Observation
of iron diffusion in the near-surface region
of magnetite at 470 K. Phys. Rev. Res..

[62] Powell C. J., Jablonski A. (2002). The NIST Electron
Effective-Attenuation-Length Database. Journal
of Surface Analysis.

[63] Lundy T. S., Coghlan W. A. (1973). Article. J. Phys. Colloques.

[64] Venkatu D. A., Poteat L. E. (1970). Diffusion of titanium of single crystal
rutile. Mater. Sci. Eng.

[65] Lee D. K., Yoo H. I. (2006). Unusual oxygen re-equilibration
kinetics of TiO_2−δ_. Solid State Ionics.

[66] Zhang Z., Lee J., Yates J. T., Bechstein R., Lira E., Hansen J. Ø., Wendt S., Besenbacher F. (2010). Unraveling the Diffusion of Bulk
Ti Interstitials in Rutile TiO_2_(110) by Monitoring Their
Reaction with O Adatoms. J. Phys. Chem. C.

