# Supplementary material for: Pt Particles on a Dynamic TiO2 Support in Near-Ambient Conditions–Disentangling Size, Pressure, and Support Effects
Source: J Am Chem Soc. 2025 Oct 17;147(43):39846–59. doi: 10.1021/jacs.5c14353 (PMC12576813; doi:10.1021/jacs.5c14353)
Supplement: Supplementary file 1 [file ja5c14353_si_001.pdf]

Supporting Information

Pt particles on a dynamic TiO_2 support in near-ambient conditions – disentangling size, pressure, and support effects

Florian Kraushofer,[1] Matthias Krinninger,[1] Marina de la Higuera-Domingo,[1] Lorenz Falling,[1] Lukas Strauss,[1] Sebastian Kaiser,[1] Mohammad Salehi,[1] Gaurav Anand,[1] Virginia Pérez Dieste,[2] Monika Blum,[3] Barbara A. J. Lechner*,[1,4]

[1] Functional Nanomaterials Group and Catalysis Research Center, Department of Chemistry, TUM School of Natural Sciences, Technical University of Munich, 85748 Garching, Germany.

[2] ALBA Synchrotron Light Source, Carrer de la Llum 2-26, Cerdanyola del Vallès, 08290 Barcelona, Spain.

[3] Chemical Sciences Division and Advanced Light Source, Lawrence Berkeley National Laboratory, Berkeley, CA 94720, USA

[4] Institute for Advanced Study, Technical University of Munich, 85748 Garching, Germany.

*Corresponding author: bajlechner@tum.de

SESSA simulations for Pt coverage quantification in XPS

During NAP-XPS experiments at synchrotrons, the Pt deposition was initially only controlled by evaporator flux. To obtain actual coverages, we performed simulations using SESSA[1, 2] to obtain Pt 4*f* / Ti 3*s* peak area ratios and compare to experiment. This requires some assumptions about the morphology of the samples, in particular the spacing and shape of the Pt particles. Since we had STM data available, we used the actually observed mean cluster densities. The amount of Pt deposited per area was precisely calibrated by QCM in the STM experiments, allowing us to construct and test a model for particle morphology, which we then applied to the NAP-XPS experiments where we don't have as much detailed information. For the shape, we found that a simple 2D island model, using only the radius and height of Pt clusters as free parameters, represents our samples most closely.

Two different approaches were tested: First, we fixed the heights of the particles to the mean apparent heights observed in STM, then calculated the particle radii based on the deposited amount of Pt, assuming bulk fcc Pt density. Using the measured cluster density and mean apparent height for 0.2 ML Pt on LR-TiO_2 as measured in the experiment shown in Figure S1 (a), this yields an expected Pt 4*f* / Ti 3*s* area ratio of 1.37, in excellent agreement with the XPS data shown in Figure 2 (c). For 0.05 ML Pt on HR-TiO_2, cluster density and mean apparent height related to Figure 2 (e) would predict a Pt 4*f* / Ti 3*s* area ratio of 0.36, somewhat lower than observed, suggesting that slightly more Pt was deposited in the XPS experiments than in the corresponding STM data.

In a second approach, we instead ignore the apparent height from STM, which may also carry a systematic error due to electronic effects. Instead, we use only the particle density, and assume that the thickness should be a multiple of the Pt(111) step height (2.266 Å). Note that this is a very rough approximation for sub-nanometer clusters, which do not follow bulk-like layer stacking. The particle radius then again follows from the bulk fcc Pt density. This approach gives a Pt 4*f* / Ti 3*s* ratio of 1.26 for 0.2 ML Pt on LR-TiO_2 assuming tri-layer clusters (6.80 Å height), or 1.42 assuming bilayer clusters (4.53 Å height), both reasonably close to the experimental data. For 0.05 ML Pt on HR-TiO_2, this

approach yields a Pt 4*f* / Ti 3*s* ratio of 0.35 assuming bilayer clusters. Tri-layer clusters would have a larger height than radius and are therefore not considered.

Overall, the difference between these two approaches is small, and the simulated intensities are in excellent agreement for 0.2 ML Pt on LR-TiO_2. The simulated Pt 4*f* intensity for 0.05 ML Pt on HR-TiO_2 is slightly smaller than that observed in the corresponding XPS experiment. Comparing to the simulations for 0.2 ML on LR-TiO_2, we can nonetheless be confident that the actual deposited amount is in the range of 0.05 to 0.1 ML. Since no coverage effects are expected in this range [compare also Figure 2 (a, b) and Figure S1], this would not affect our conclusions.

Estimating maximum TiO_2 thicknesses in oxidative layer growth

Initially setting aside kinetic considerations, the Ti_{int} concentration in bulk TiO_2 can be as high as one per 1250 unit cells, or $x \approx 4 \times 10^{-4}$ in TiO_{2-x}.[3] Since our HR-TiO_2 crystals do not yet exhibit a (1×2) surface reconstruction, the value is likely lower. Nonetheless, using $x = 4 \times 10^{-4}$ as a first approximation, it follows that complete re-oxidation can increase the thickness of the sample by at most 0.04%. The samples used in the synchrotron NAP-XPS experiments (Figures 2, 3 and 6) had a thickness of only 0.5 mm, resulting in a maximum added thickness of 200 nm if they were fully re-oxidized. The samples used for NAP-STM and the lab-based NAP-XPS measurements were hat-shaped with a total thickness of 2.3 mm, which would yield up to 920 nm growth using the same approximations.

To account for diffusion kinetics, we apply a simple random-walk model of non-interacting Ti_{int}, as described previously.[4] The probability distribution of where to find a diffusing particle after an annealing step is given by a normal distribution centered at that particles' original position with standard deviation σ, which depends on the annealing time, temperature, and diffusion barrier. We can obtain the approximate number of Ti_{int} 'impinging' on each surface unit cell from the bulk at least once during the annealing time by integrating the survival function *S*(*z*) of this normal distribution over the thickness d_S of the sample, times the initial uniform linear density ρ of Ti_{int}:

$$N_{\mathrm{Ti}} = \int_0^{d_S} \rho \cdot S(\sigma, z)\, dz$$

$$\rho = 2 \cdot x / d_L$$

with the interlayer distance d_L = 3.25 Å and Ti_{int} density $x = 4 \times 10^{-4}$. To obtain a maximum possible thickness of added TiO_2 layers, we now assume that each of the Ti atoms arriving at the surface is oxidized. The thickness then easily follows as $0.5 \cdot N_{\mathrm{Ti}} \cdot d_L$, such that the added thickness is simply

$$d_{\mathrm{add}} = x \int_0^{d_S} S(\sigma, z)\, dz$$

This expression converges to 39.3 nm for sufficiently large d_S and 15 minutes annealing at 600 K, assuming a 0.5 eV diffusion barrier.[5-7] Since σ scales with the square root of time,[4] annealing for 30 minutes instead only increases this value to 55.5 nm, and annealing for only 5 minutes still yields a possible thickness of 22.7 nm.

In addition, we can find an upper limit to the bulk Ti_{int} diffusion barrier, above which fewer Ti_{int} would reach the surface than are needed to explain the observed attenuation of the Pt signal. Taking the conservative limits of (i) no additional surface barriers (*i.e.* every Ti_{int} reaching the surface is directly

integrated into a new TiO_2 terrace), (ii) maximum bulk Ti_{int} concentration of $x = 4 \times 10^{-4}$,[3] (iii) an arbitrarily thick TiO_2 bulk (> 1 mm), and (iv) attenuation of the Pt signal to 5% of the initial intensity (requiring only 7.3 nm of TiO_2 covering the particle),[8] the highest barrier that would still fit the observed Pt 4*f* attenuation within 5 minutes at 600 K is $E_B = 0.62$ eV.

Supplementary Figures

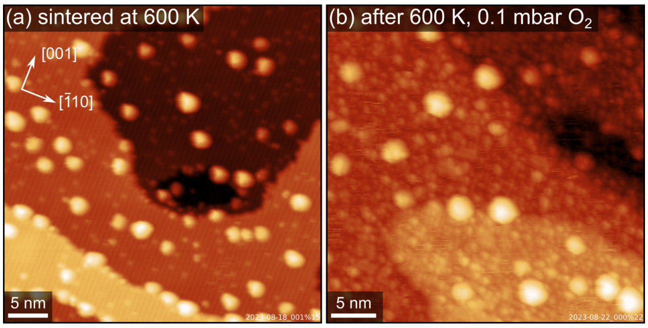

Figure S1. STM images of 0.2 ML Pt on LR-TiO_2 (a) sintered at 600 K, and (b) after 15 minutes in 0.1 mbar O_2 at 600 K. *Imaging conditions:* RT, UHV, (a) $U_b = 2.0$ V, $I_t = 0.2$ nA, (b) $U_b = 1.6$ V, $I_t = 0.2$ nA.

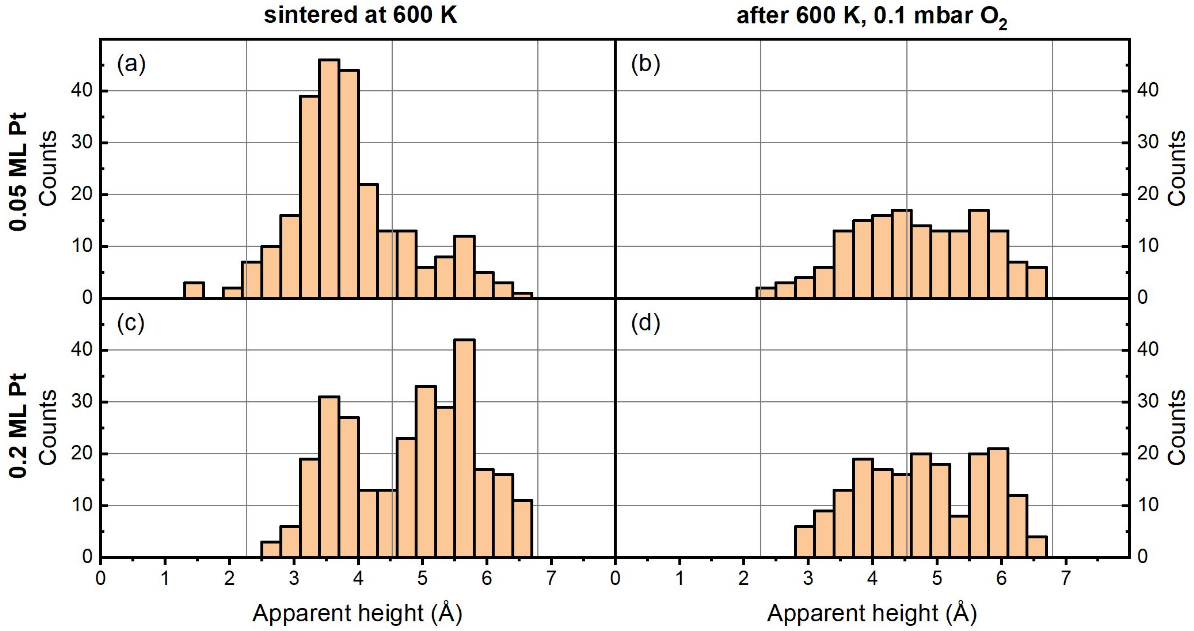

Figure S2. Apparent height distributions of Pt clusters on LR-TiO_2 as measured in STM before and after exposure to 0.1 mbar O_2, corresponding to data shown in Figure 2 (a, b) and Figure S1. Only clusters on terraces were considered. Interlayer distances for bulk Pt(111) are drawn as vertical grey lines as a guide to the eye.

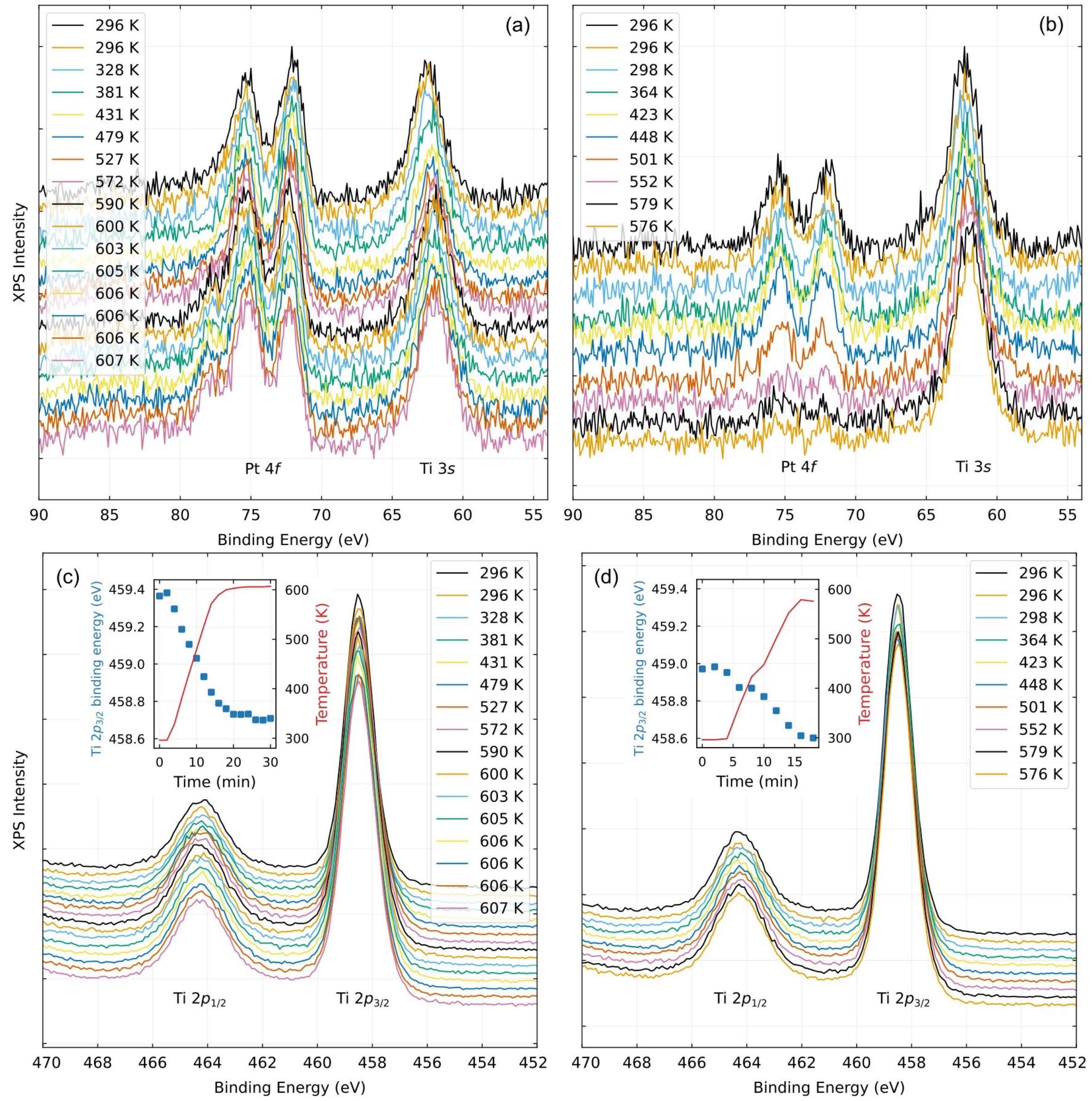

Figure S3. NAP-XPS spectra (*hν* = 650 eV, normalized to Ti 3*s* peak) of the Pt 4*f* and Ti 3*s* region for Pt on (a) LR-TiO_2 and (b) HR-TiO_2, acquired in 0.1 mbar O_2 while heating from room temperature to 600 K. Subsets of the same data are shown in Figure 2 (c) and (g), smoothed with a 3-point running average filter for clarity. The raw data as shown here was used to obtain the integrated peak area ratios in Figure 2 (d) and (h). (c) and (d) show Ti 2*p* spectra from the same experiments. The energy scale in (c) and (d) is calibrated to keep the Ti $2p_{3/2}$ component consistently at 458.5 eV, allowing direct comparison of peak shapes. Ti $2p_{3/2}$ positions of the raw data as a function of temperature are shown in the insets. The same trends can be seen in the raw Ti 3*s* peaks in panels (a) and (b), as well as the O 1*s* peaks, as discussed in the main manuscript.

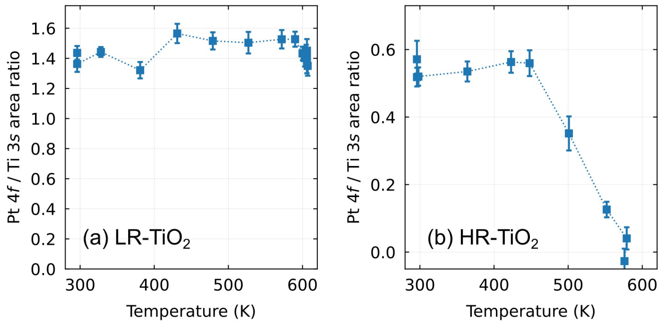

Figure S4: Area ratios of integrated Pt 4*f* and Ti 3*s* peaks corresponding to the data in Figure S3 as a function of temperature. The data points for Pt (a) LR-TiO_2 and (b) HR-TiO_2 are the same as the ones shown as a function of time in Figure 2 (d) and (h), respectively. Dotted lines are drawn to guide the eye and connect data points in order of acquisition.

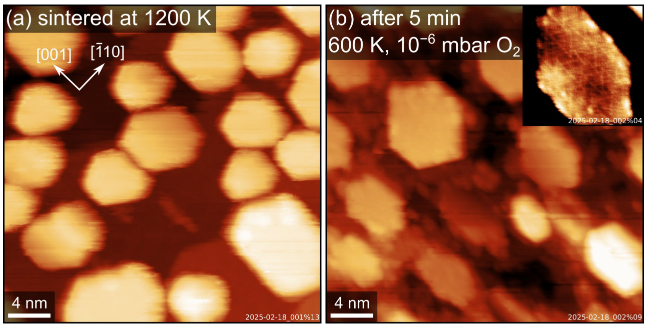

Figure S5. STM images of Pt nanoparticles on HR-TiO_2 (a) sintered at 1200 K in UHV, then (b) annealed for 5 minutes at 600 K in 10^{-6} mbar O_2. A subsequent higher-resolution image revealing the superstructure on the top facet of a nanoparticle is shown in the inset. *Imaging conditions:* RT, UHV, (a) U_b = 1.5 V, I_t = 0.1 nA, (b) U_b = 1.9 V, I_t = 0.8 nA, and (inset) U_b = 1.9 V, I_t = 0.2 nA.

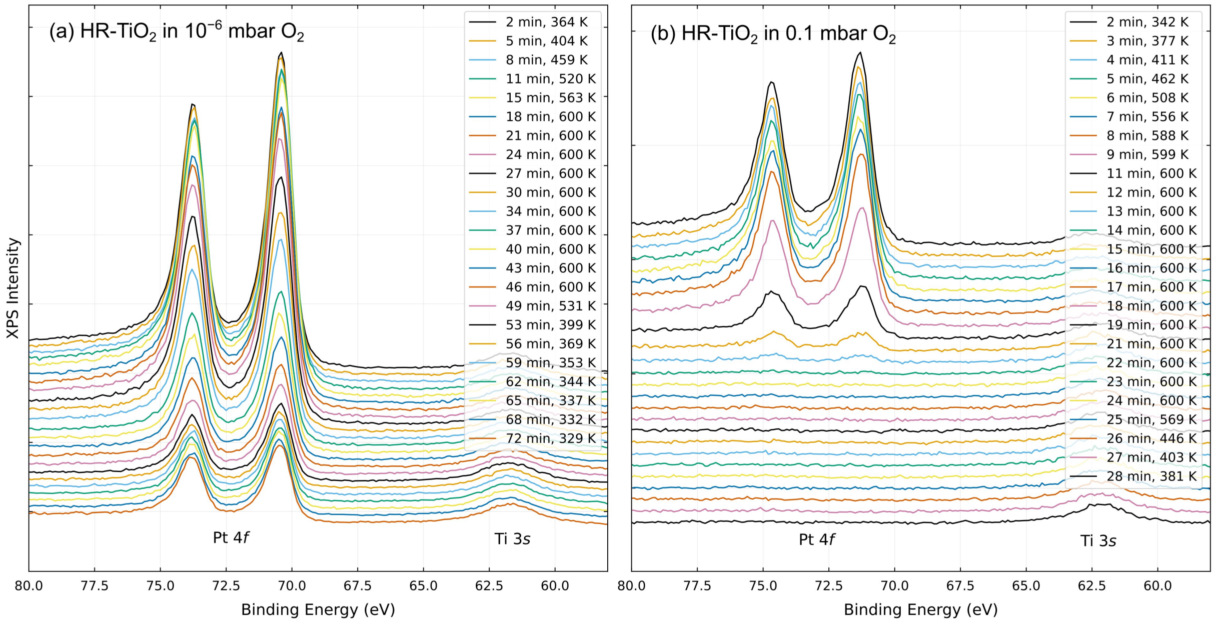

Figure S6. NAP-XPS spectra (monochromatic Al Kα, normalized to Ti 3*s* peak) of the Pt 4*f* and Ti 3*s* region for Pt on HR-TiO_2 in (a) 10^{-6} mbar O_2 and (b) 0.1 mbar O_2, acquired while annealing to 600 K. Each spectrum in (a) and (b) corresponds to one peak area ratio data point in Figure 4 (i) and the inset to Figure 5 (e), respectively.

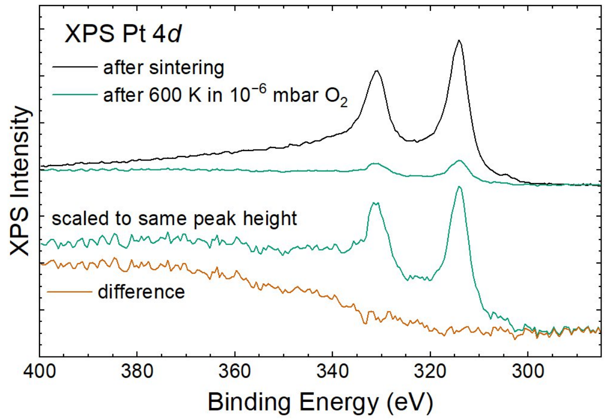

Figure S7. Pt 4*d* XPS spectra (monochromatic Al Kα) for Pt nanoparticles on HR-TiO_2 before (black) and after (green) annealing at 600 K in 10^{-6} mbar O_2. Data was acquired in the same experiment as the spectra shown in Figure 4 (g, h). (top) Both spectra are normalized to the low-binding-energy background, showing the same trend of decreased Pt peak area after O_2 annealing as seen in Figure 4. (bottom) The spectrum after O_2 annealing (green) is scaled to the same peak height as the one before (black) to allow direct comparison of the background shape. The difference between the black and the scaled green line is drawn in orange.

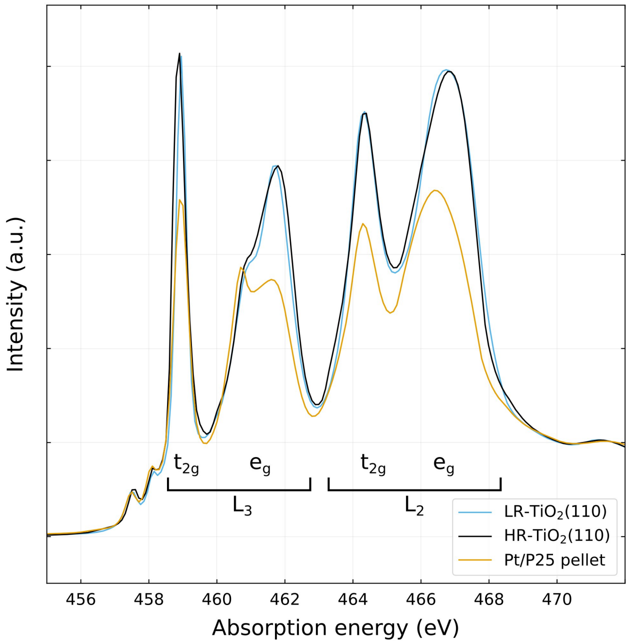

Figure S8. NEXAFS spectra of the Ti $L_{2,3}$ edge comparing samples investigated by NAP-XPS in Figure 6. Spectra on TiO_2 single crystals were acquired after sample cleaning in UHV, but before Pt deposition. The spectrum on the Pt/P25 pellet was acquired in 1 mbar H_2 before heating. No substantial change in the NEXAFS peak shapes was observed in the course of the experiments for any of the samples, as shown in Figure S9.

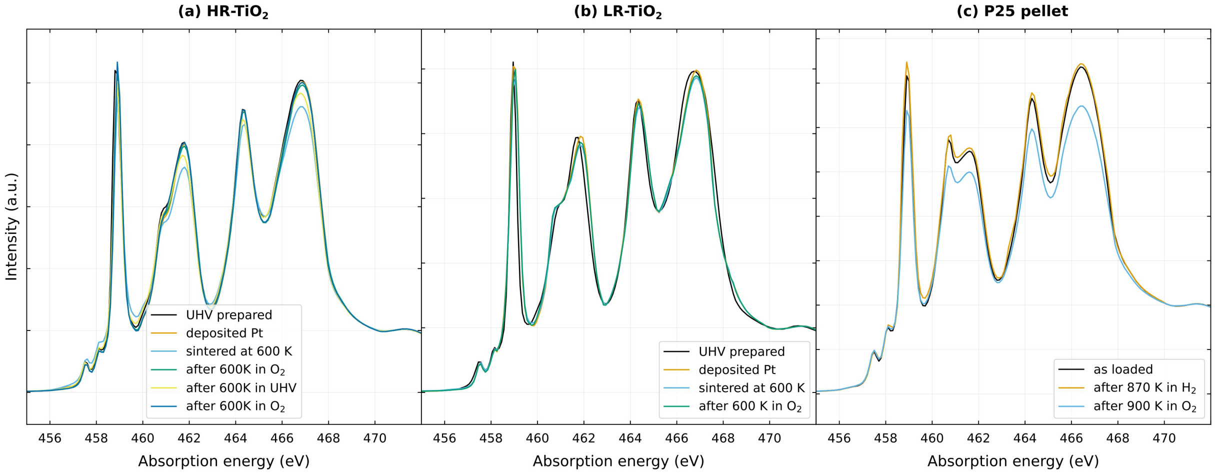

Figure S9. Evolution of NEXAFS Ti $L_{2,3}$ edge spectra over the course of the experiments in Figure 6 for (a) HR-TiO_2, (b) LR-TiO_2, and (c) the Pt-loaded P25 pellet.

References

1. Smekal, W.; Werner, W. S. M.; Powell, C. J., Simulation of electron spectra for surface analysis (SESSA): a novel software tool for quantitative Auger-electron spectroscopy and X-ray photoelectron spectroscopy. *Surf. Interface Anal.* **2005,** *37* (11), 1059-1067, DOI: 10.1002/sia.2097.
2. Werner, W.; Smekal, W.; Powell, C. J., NIST Database for the Simulation of Electron Spectra for Surface Analysis (SESSA), SRD 100, Version 2.1. 2017.
3. Aono, M.; Hasiguti, R. R., Interaction and ordering of lattice defects in oxygen-deficient rutile TiO_{2-x}. *Phys. Rev. B* **1993,** *48* (17), 12406-12414, DOI: 10.1103/PhysRevB.48.12406.
4. Kraushofer, F.; Krinninger, M.; Kaiser, S.; Reich, J.; Jarosz, A.; Füchsl, M.; Anand, G.; Esch, F.; Lechner, B. A. J., The influence of bulk stoichiometry on near-ambient pressure reactivity of bare and Pt-loaded rutile TiO_2(110). *Nanoscale* **2024,** *16* (38), 17825-17837, DOI: 10.1039/d4nr01702a.
5. Lundy, T., S.; Coghlan, W., A., Cation self diffusion in rutile. *J. Phys. Colloques* **1973,** *34* (C9), C9-299-C9-302, DOI: 10.1051/jphyscol:1973953.
6. Venkatu, D. A.; Poteat, L. E., Diffusion of titanium of single crystal rutile. *Materials Science and Engineering* **1970,** *5* (5), 258-262, DOI: 10.1016/0025-5416(70)90014-5.
7. Lee, D. K.; Yoo, H. I., Unusual oxygen re-equilibration kinetics of $TiO_{2-\delta}$. *Solid State Ionics* **2006,** *177* (1), 1-9, DOI: 10.1016/j.ssi.2005.10.016.
8. Powell, C. J.; Jablonski, A., The NIST Electron Effective-Attenuation-Length Database. *Journal of Surface Analysis* **2002,** *9* (3), 322-325, DOI: 10.1384/jsa.9.322.
